# Supplementary material for: Humoral and Cellular Immunity following Five Doses of COVID-19 Vaccines in Solid Organ Transplant Recipients: A Systematic Review and Meta-Analysis
Source: Vaccines (Basel). 2023 Jun 27;11(7):1166. doi: 10.3390/vaccines11071166 (PMC10384009; doi:10.3390/vaccines11071166)

Risk of Bias Assessment (Observational studies):

	Study	Selection				Comparability	Outcome			Score
		Representativeness of the exposed cohort	Selection of the non-exposed cohort	Ascertainment of exposure	Demonstration that outcome of interest was not present at start of study	Comparability of cohorts on the basis of the design or analysis	Assessment of outcome	Was follow-up long enough for outcomes to occur	Adequacy of follow up of cohorts	
1	Yael Peled, MD, August 2021	1	0	1	1	0	1	1	1	6
2	Brian J. Boyarsky, August 2021	1	1	1	1	1	1	0	0	6
3	Schmidt T, et al., preprint May, 2021	1	1	1	1	1	1	1	1	8
4	Grupper et al, April, 2021	1	1	1	1	1	1	1	1	8
5	Rabinowich L, et al. April 2021	1	1	1	1	1	1	1	1	8
6	Korth J et al. April ,2021	1	1	1	1	1	1	1	1	8
7	Marinaki et al, April, 2021	1	1	1	1	1	1	1	1	8
8	Jackrapong Bruminhent, et al., October 2021	1	1	1	1	1	1	0	0	6
9	Ahmad Yanis, Et,al., November 2021	1	1	1	1	1	1	1	1	8
10	Louise Benning, Et,al., jan 2022	1	1	1	1	1	1	1	1	8
11	Michael T. Ou, Et, al., Sep 2021	1	1	1	1	1	1	1	1	8
12	Andrew H. Karaba, Et, al., Dec 2021	1	1	1	1	1	1	1	1	8
13	René Schramm, Et,al., July 2021	1	0	1	1	1	1	1	1	7
14	Alessandra Mazzola, Et, al., june 2021	1	0	1	1	1	1	1	1	7
15	Ana Luísa Correia, Et, al., jan 2022	1	1	1	1	1	1	1	1	8
16	Çiğdem Erol,Et, al., Dec 2021	1	0	1	1	1	1	1	1	7
17	Sebastian Rask Hamm,Et,al., Feb 2022	1	1	1	1	1	1	1	1	8
18	Gianpiero D'Offizi, Et, al., Oct 2021	1	0	1	1	1	1	1	1	7
19	Yana Davidov,Et,al., oct 2021	1	1	1	1	1	1	1	1	8
20	Tammy Hod,Et,al., Nov 2021	1	1	1	1	1	1	1	1	8
21	Marta Kantauskaite, Et, al., Feb 2022	1	1	1	1	1	1	1	1	8
22	Mariana Seija, Et, al., Dec 2021	1	1	1	1	1	1	1	1	8

Risk of Bias Assessment (RCTs)

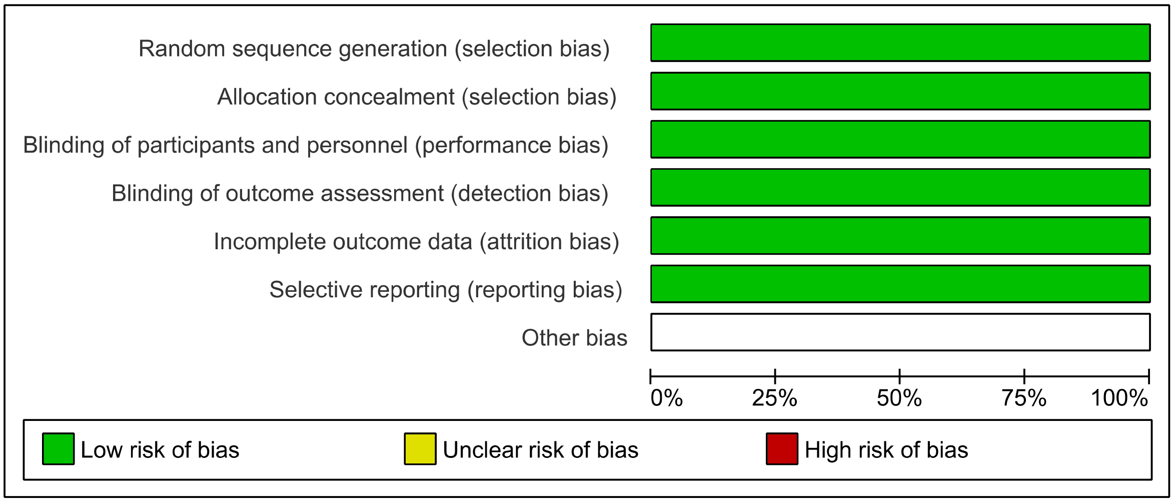

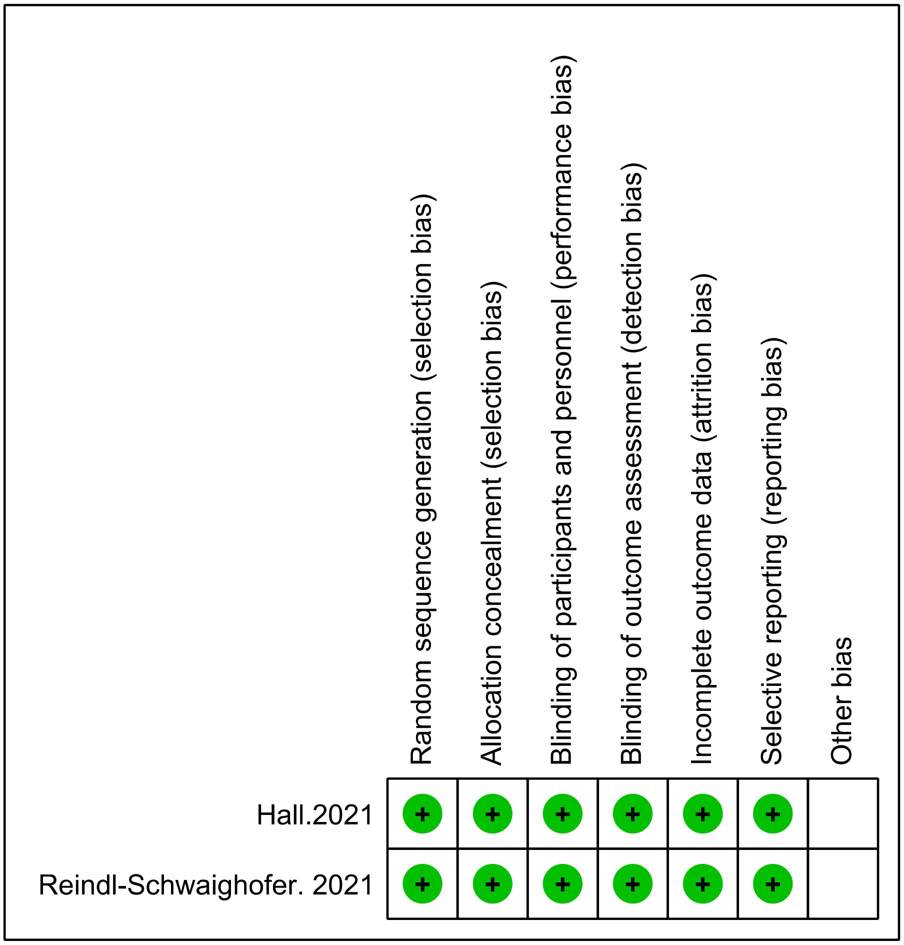

Supplement: Supplementary file 1 [file vaccines-11-01166-s001.zip › File S4 Risk of Bias Assessment.pdf]
