# Supplementary material for: Humoral and Cellular Immunity following Five Doses of COVID-19 Vaccines in Solid Organ Transplant Recipients: A Systematic Review and Meta-Analysis
Source: Vaccines (Basel). 2023 Jun 27;11(7):1166. doi: 10.3390/vaccines11071166 (PMC10384009; doi:10.3390/vaccines11071166)
Supplement: Supplementary file 1 [file vaccines-11-01166-s001.zip › File S5 Doses Analysis.pdf]

Figure (S1): Pooled proportion of +ve antispike Ab after the first dose administration:

Pooled proportion of positive antispike Ab after the 1st dose of vaccination

Study name	Subgroup within study	Event rate	Lower limit	Upper limit	Total	Relative weight
Strauss, 2021	After 1st Dose	0.342	0.273	0.418	55 / 161	6.60
Kamar, 2021	After 1st Dose	0.040	0.015	0.101	4 / 101	5.68
Hall, 2021	After 1st Dose	0.050	0.022	0.106	6 / 121	5.99
Herrera, 2021	After 1st Dose	0.260	0.184	0.352	27 / 104	6.50
Boyarsky (A), 2021	After 1st Dose	0.149	0.124	0.178	98 / 658	6.67
Boyarsky (B), 2021	After 1st Dose	0.586	0.550	0.621	432 / 737	6.70
Shostak, 2021	After 1st Dose	0.036	0.016	0.077	6 / 168	6.00
Ben Zadok, 2021	After 1st Dose	0.154	0.071	0.303	6 / 39	5.91
Schmidt, 2021	After 1st Dose	0.053	0.013	0.187	2 / 38	4.90
Chavarot, 2021	After 1st Dose	0.020	0.005	0.076	2 / 101	4.95
Benotmane, 2021	After 1st Dose	0.122	0.084	0.174	25 / 205	6.52
Boyarsky, (C), 2021	After 1st Dose	0.174	0.142	0.213	76 / 436	6.66
Yanis 2021	After 1st Dose	0.010	0.001	0.136	0 / 51	2.77
Ou 2021	After 1st Dose	0.130	0.105	0.160	77 / 592	6.66
Schramm 2021	After 1st Dose	0.040	0.010	0.146	2 / 50	4.92
Mazzola 2021	After 1st Dose	0.072	0.038	0.133	9 / 125	6.21
Erol 2021	After 1st Dose	0.479	0.343	0.618	23 / 48	6.36
		0.119	0.069	0.198	850 / 3735	

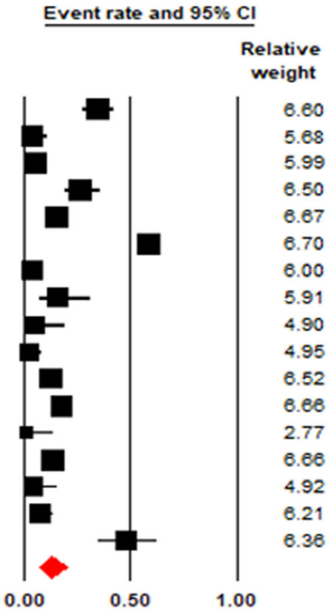

Heterogeneity: τ^2=1.42; Q=611.4, df=16, P<0.001; I^2=97%

#Studies: 17 #Patients: 3735 #Response: 850
#Proportion (95%CI): 0.12 (0.07 to 0.2)

The pooled proportion of positive antispike Ab after the first dose of vaccination from the random effects model is 0.12 (95%CI: 0.07 to 0.2). There is a considerable heterogeneity between studies reporting seroconversion after administration of the second dose of the vaccine (I^2 = 81.6%).

Table (S1): sensitivity analysis showing the results after one study removal from the 17 studies used in the above forest plot:

Studies	Statistics with study removed			
	Estimate	Lower limit	Upper limit	p-Value
Strauss, 2021	0.108	0.059	0.189	< 0.001
Kamar, 2021	0.127	0.073	0.212	< 0.001
Hall, 2021	0.126	0.072	0.210	< 0.001
Herrera, 2021	0.111	0.062	0.192	< 0.001
Boyarsky (A), 2021	0.116	0.063	0.202	< 0.001
Boyarsky (B), 2021	0.116	0.082	0.161	< 0.001
Shostak, 2021	0.128	0.074	0.214	< 0.001
Ben Zadok, 2021	0.117	0.066	0.198	< 0.001
Schmidt, 2021	0.124	0.071	0.208	< 0.001
Chavarot, 2021	0.130	0.075	0.216	< 0.001
Benotmane, 2021	0.118	0.067	0.202	< 0.001
Boyarsky, (C), 2021	0.114	0.062	0.200	< 0.001
Yanis 2021	0.127	0.073	0.211	< 0.001
Ou 2021	0.117	0.065	0.202	< 0.001
Schramm 2021	0.125	0.072	0.210	< 0.001
Mazzola 2021	0.123	0.070	0.207	< 0.001
Erol 2021	0.106	0.059	0.182	< 0.001
Random	0.119	0.069	0.198	< 0.001

This table of sensitivity analysis indicates that the results are robust in the sense that the pooled seroconversion after the first dose of vaccination will remain essentially the same (range from 10.6% to 13%) with any one study removed.

Figure (S2) funnel plot of the 17 studies showing the immune response after the first dose:

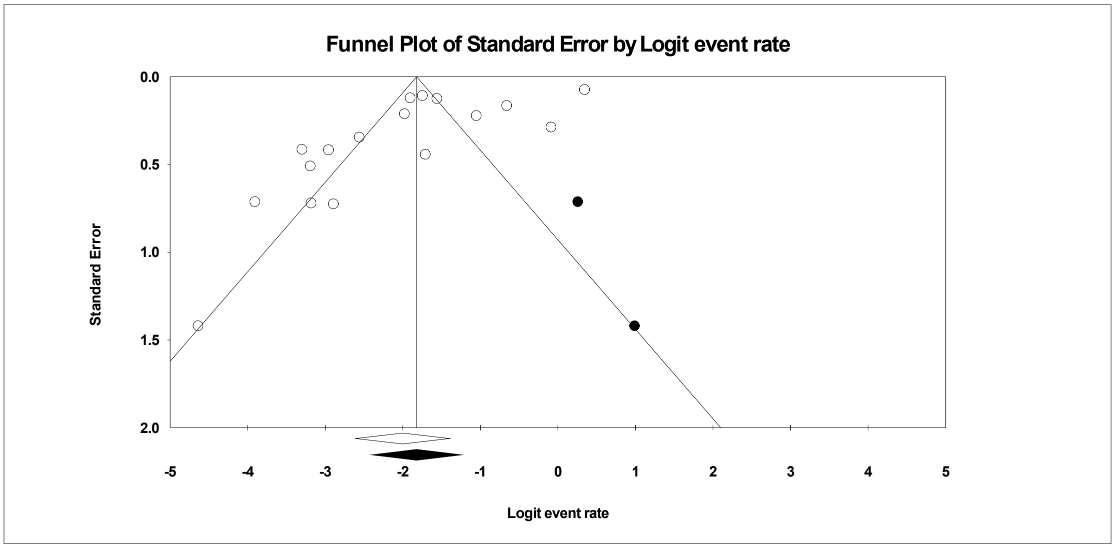

Egger's regression intercept

Intercept	-5.58311
Standard error	2.06943
95% lower limit (2-tailed)	-9.99400
95% upper limit (2-tailed)	-1.17223
t-value	2.69790
df	15.00000
P-value (1-tailed)	0.00826
P-value (2-tailed)	0.01653

Duval and Tweedie's trim and fill

		Fixed Effects			Random Effects			Q Value
	Studies Trimmed	Point Estimate	Lower Limit	Upper Limit	Point Estimate	Lower Limit	Upper Limit	
**Observed values**		0.27674	0.25982	0.29432	0.11886	0.06861	0.19807	611.36414
**Adjusted values**	2	0.27804	0.26111	0.29562	0.13919	0.08284	0.22448	616.15791

There is evidence of publication bias in the pooled analysis of seroconversion after the first dose of vaccination. There are two studies resided at the left of the funnel are more than those on the right side. The Egger's regression intercept (B0) is -5.58, (95%CI: -9.99, -1.17), with t=2.7, df=15. The 1-tailed p-value (recommended) is 0.00826, and the 2-tailed p-value is 0.01653.; indicating the possibly of publication bias.

Using Trim and Fill method, the adjusted pooled proportion (random effect) of seroconversion after the second dose of vaccination would increase from to 0.12 to 0.14 (95% CI: 0.08 to 0.22) if the publication bias was not existed.

Figure (S3): Pooled proportion of +ve antispike Ab after the second dose administration:

Pooled proportion of positive antispike Ab after the 2nd dose of vaccination

Study name	Subgroup within study	Event rate	Lower limit	Upper limit	Total	Relative weight
Strauss, 2021	After 2nd Dose	0.466	0.390	0.543	75 / 161	4.51
Kamar, 2021	After 2nd Dose	0.364	0.275	0.463	36 / 99	4.14
Peled, 2021	After 2nd Dose	0.182	0.111	0.284	14 / 77	3.48
Hall, 2021	After 2nd Dose	0.291	0.214	0.382	32 / 110	4.13
Herrera, 2021	After 2nd Dose	0.385	0.296	0.481	40 / 104	4.20
Boyarsky (A), 2021	After 2nd Dose	0.394	0.357	0.431	259 / 658	4.94
Shostak, 2021	After 2nd Dose	0.181	0.125	0.254	25 / 138	4.05
Rozen-Zvi , 2021	After 2nd Dose	0.364	0.312	0.419	112 / 308	4.75
Ben Zadok, 2021	After 2nd Dose	0.357	0.204	0.546	10 / 28	2.78
Schmidt, 2021	After 2nd Dose	0.294	0.166	0.466	10 / 34	2.90
Grupper, 2021	After 2nd Dose	0.375	0.298	0.459	51 / 136	4.37
Rabinowich, 2021	After 2nd Dose	0.475	0.368	0.584	38 / 80	4.03
Korth, 2021	After 2nd Dose	0.217	0.093	0.428	5 / 23	2.15
Chavarot, 2021	After 2nd Dose	0.057	0.014	0.202	2 / 35	1.33
Benotmane, 2021	After 2nd Dose	0.361	0.298	0.429	74 / 205	4.59
Marinaki, 2021	After 2nd Dose	0.588	0.419	0.739	20 / 34	3.09
Bruminhent 2021	After 2nd Dose	0.114	0.044	0.268	4 / 35	2.03
Alejo 2021	After 2nd Dose	0.278	0.121	0.519	5 / 18	2.05
Peled 2021	After 2nd Dose	0.271	0.191	0.368	26 / 96	3.98
Schrezenmeier 2021	After 2nd Dose	0.019	0.001	0.244	0 / 25	0.43
Holden 2021	After 2nd Dose	0.350	0.254	0.460	28 / 80	3.95
Yanis 2021	After 2nd Dose	0.216	0.124	0.349	11 / 51	3.15
Sadioglu 2021	After 2nd Dose	0.188	0.119	0.285	16 / 85	3.61
Benning 2022	After 2nd Dose	0.304	0.232	0.386	41 / 135	4.30
Ou 2021	After 2nd Dose	0.478	0.429	0.527	191 / 400	4.85
Karaba 2021	After 2nd Dose	0.234	0.135	0.375	11 / 47	3.12
Schramm 2021	After 2nd Dose	0.060	0.019	0.170	3 / 50	1.76
Mazzola 2021	After 2nd Dose	0.218	0.156	0.296	29 / 133	4.13
Erol 2021	After 2nd Dose	0.250	0.148	0.390	12 / 48	3.20
		0.301	0.262	0.342	1180 / 3433	

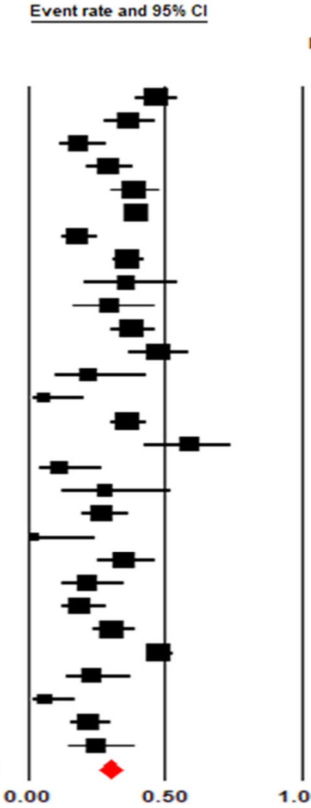

Heterogeneity: τ^2=0.19; Q=152, df=28, P<0.001; I^2=81.6%

#Studies: 29 #Patients: 3433 #Response: 1180
#Proportion (95%CI): 0.30 (0.26 to 0.34)

The pooled proportion of positive antispike Ab after the second dose of vaccination from the random effects model is 0.3 (95%CI: 0.26 to 0.34; N = 3433). There is a substantial heterogeneity between studies reporting seroconversion after administration of the second dose of the vaccine (I^2 = 81.6%). Worth to notice that the seroconversion was increased from 12% after the first dose to 30% after the second dose.

Table (S2): sensitivity analysis showing the results after one study removal from the 29 studies used in the above forest plot:

Study name	Statistics with study removed			
	Point	Lower limit	Upper limit	p-Value
Strauss, 2021	0.29	0.25	0.336	< 0.001
Kamar, 2021	0.30	0.26	0.341	< 0.001
Peled, 2021	0.31	0.27	0.348	< 0.001
Hall, 2021	0.30	0.26	0.344	< 0.001
Herrera, 2021	0.30	0.26	0.340	< 0.001
Boyarsky (A), 2021	0.29	0.25	0.339	< 0.001
Shostak, 2021	0.31	0.27	0.350	< 0.001
Rozen-Zvi , 2021	0.30	0.26	0.340	< 0.001
Ben Zadok, 2021	0.30	0.26	0.341	< 0.001
Schmidt, 2021	0.30	0.26	0.343	< 0.001
Grupper, 2021	0.30	0.26	0.340	< 0.001
Rabinowich, 2021	0.29	0.26	0.336	< 0.001
Korth, 2021	0.30	0.26	0.345	< 0.001
Chavarot, 2021	0.31	0.27	0.348	< 0.001
Benotmane, 2021	0.30	0.26	0.340	< 0.001
Marinaki, 2021	0.29	0.26	0.335	< 0.001
Bruminhent 2021	0.31	0.27	0.348	< 0.001
Alejo 2021	0.30	0.26	0.343	< 0.001
Peled 2021	0.30	0.26	0.345	< 0.001
Schrezenmeier 2021	0.30	0.27	0.345	< 0.001
Holden 2021	0.30	0.26	0.341	< 0.001
Yanis 2021	0.30	0.26	0.346	< 0.001
Sadioglu 2021	0.31	0.27	0.348	< 0.001
Benning 2022	0.30	0.26	0.343	< 0.001
Ou 2021	0.29	0.26	0.335	< 0.001
Karaba 2021	0.30	0.26	0.346	< 0.001
Schramm 2021	0.31	0.27	0.350	< 0.001
Mazzola 2021	0.31	0.27	0.348	< 0.001
Erol 2021	0.30	0.26	0.345	< 0.001
Random	0.30	0.26	0.342	< 0.001

This table of sensitivity analysis indicates that the results are robust in the sense that the pooled seroconversion after the second dose of vaccination will remain essentially the same (range from 29% to 31%) with any one study removed.

Figure (S4) funnel plot of the 29 studies showing the immune response after the second dose:

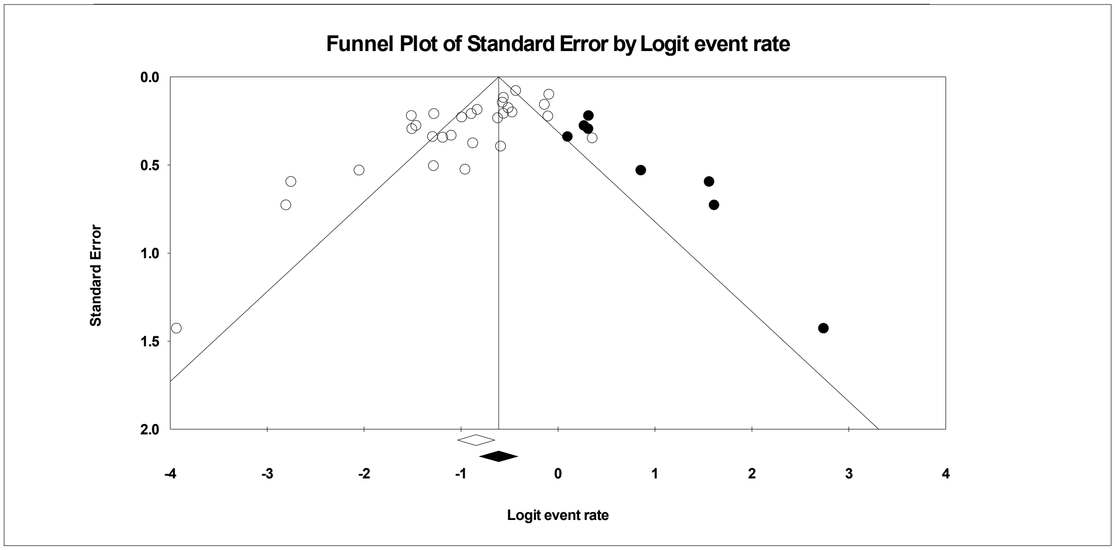

Egger's regression intercept

Intercept	-2.94099
Standard error	0.64939
95% lower limit (2-tailed)	-4.27343
95% upper limit (2-tailed)	-1.60855
t-value	4.52883
df	27.00000
P-value (1-tailed)	0.00005
P-value (2-tailed)	0.00011

Duval and Tweedie's trim and fill

		Fixed Effects			Random Effects			Q Value
	Studies Trimmed	Point Estimate	Lower Limit	Upper Limit	Point Estimate	Lower Limit	Upper Limit	
**Observed values**		0.35755	0.34112	0.37433	0.30072	0.26213	0.34235	151.96234
**Adjusted values**	8	0.37586	0.35972	0.39228	0.35153	0.30832	0.39732	221.62012

There is evidence of publication bias in the pooled analysis of seroconversion after the second dose of vaccination. There are eight studies resided at the left of the

funnel are more than those on the right side. The Egger's regression intercept (B0) is -2.94099, (95%CI: -4.27343 to -1.60855), with t=4.53 df=27. The 1-tailed p-value (recommended) is 0.00005, and the 2-tailed p-value is 0.00011; indicating the possibly of publication bias.

Using Trim and Fill method, the adjusted pooled proportion (random effect) of seroconversion after the second dose of vaccination would increase from to 0.3 to 0.38 (95% CI: 0.31 to 0.4) if the publication bias was not existed.

Figure (S5): Pooled proportion of +ve antispike Ab after the third dose administration:

Pooled proportion of positive antispike Ab after the 3rd dose of vaccination

Study name	Subgroup within study	Event rate	Lower limit	Upper limit	Total	Relative weight
Kamar, 2021	After 3rd Dose	0.263	0.185	0.358	26 / 99	9.45
Hall, (A) 2021	After 3rd Dose	0.433	0.315	0.560	26 / 60	9.09
Werbel, 2021	After 3rd Dose	0.267	0.139	0.450	8 / 30	7.28
Alejo 2021	After 3rd Dose	0.389	0.198	0.621	7 / 18	6.49
Reindl-Schwaighofer 2021	After 3rd Dose	0.386	0.320	0.456	76 / 197	10.24
Peled 2021	After 3rd Dose	0.396	0.303	0.497	38 / 96	9.66
Schrezenmeier 2021	After 3rd Dose	0.360	0.199	0.560	9 / 25	7.24
Kamar 2021	After 3rd Dose	0.135	0.057	0.286	5 / 37	6.51
Karaba 2021	After 3rd Dose	0.489	0.351	0.629	23 / 47	8.72
Kumar 2022	After 3rd Dose	0.765	0.630	0.861	39 / 51	8.27
Correia 2022	After 3rd Dose	0.580	0.494	0.662	76 / 131	9.97
Karaba, 2022	After 3rd Dose	0.680	0.478	0.831	17 / 25	7.10
		0.426	0.337	0.521	350 / 816	

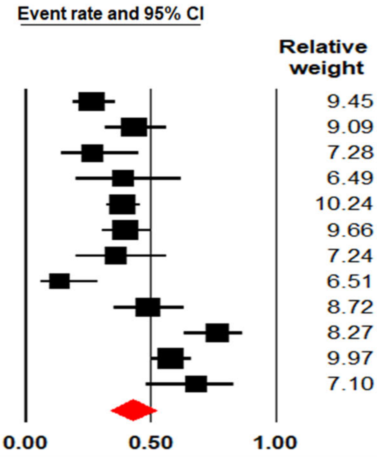

Heterogeneity: τ^2=0.35; Q=65, df=11, P<0.001; I^2=83.1%

#Studies: 12 #Patients: 816 #Response: 350
#Proportion (95%CI): 0.43 (0.34 to 0.52)

The pooled proportion of positive antispike Ab after the third dose of vaccination from the random effects model is 0.43 (95%CI: 0.34 to 0.52; N = 816). There is a substantial heterogeneity between studies reporting seroconversion after administration of the third dose of the vaccine (I^2 = 83.3%).

Table (S3): sensitivity analysis showing the results after one study removal from the 12 studies used in the above forest plot:

Study name	Statistics with study removed			
	Point	Lower limit	Upper limit	p-Value
Kamar, 2021	0.45	0.35	0.54	0.27
Hall, (A) 2021	0.43	0.33	0.53	0.16
Werbel, 2021	0.44	0.35	0.54	0.23
Alejo 2021	0.43	0.33	0.53	0.16
Reindl-Schwaighofer 2021	0.43	0.33	0.54	0.21
Peled 2021	0.43	0.33	0.54	0.19
Schrezenmeier 2021	0.43	0.34	0.53	0.18
Kamar 2021	0.45	0.36	0.54	0.31
Karaba 2021	0.42	0.32	0.52	0.13
Kumar 2022	0.40	0.32	0.48	0.02
Correia 2022	0.41	0.32	0.51	0.07
Karaba, 2022	0.41	0.32	0.50	0.06
Random	0.43	0.34	0.52	0.13

This table of sensitivity analysis indicates that the results are robust in the sense that the pooled seroconversion after the third dose of vaccination will remain essentially the same (range from 40% to 45%) with any one study removed.

Figure (S6) funnel plot of the 12 studies showing the immune response after the third dose:

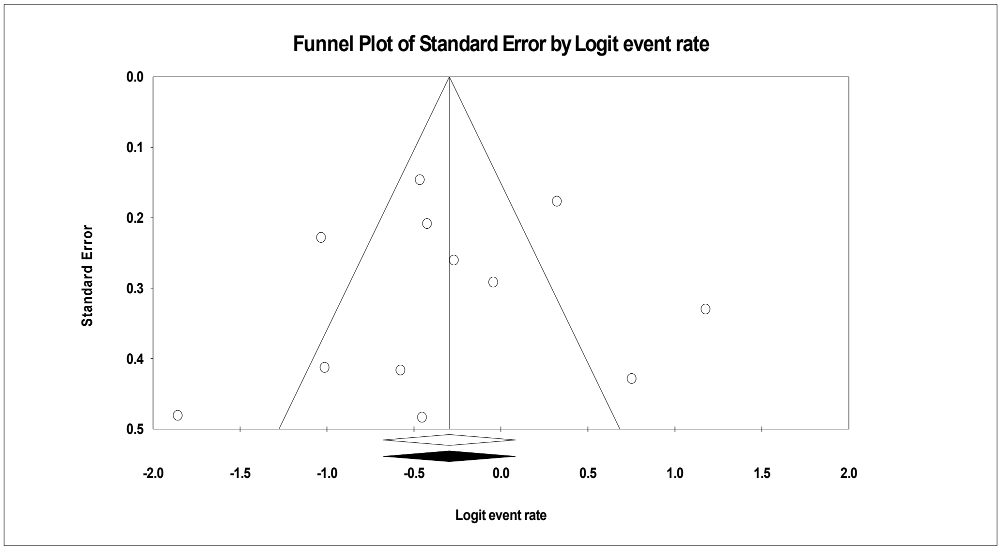

Egger's regression intercept

Intercept	-0.33503
Standard error	1.93866
95% lower limit (2-tailed)	-4.65463
95% upper limit (2-tailed)	3.98457
t-value	0.17281
df	10.00000
P-value (1-tailed)	0.43312
P-value (2-tailed)	0.86625

According to the funnel plot, where the pooled studies symmetry around the mean effect size, and Egger's regression intercept test ($P > 0.05$), there is no evidence of publication bias in this analysis. Moreover, with the random effects model the point estimate and 95% confidence interval for the pooled studies using Duval and Tweedie's Trim and Fill are unchanged.

Figure (S7): Pooled proportion of +ve antispike Ab after the fourth dose administration:

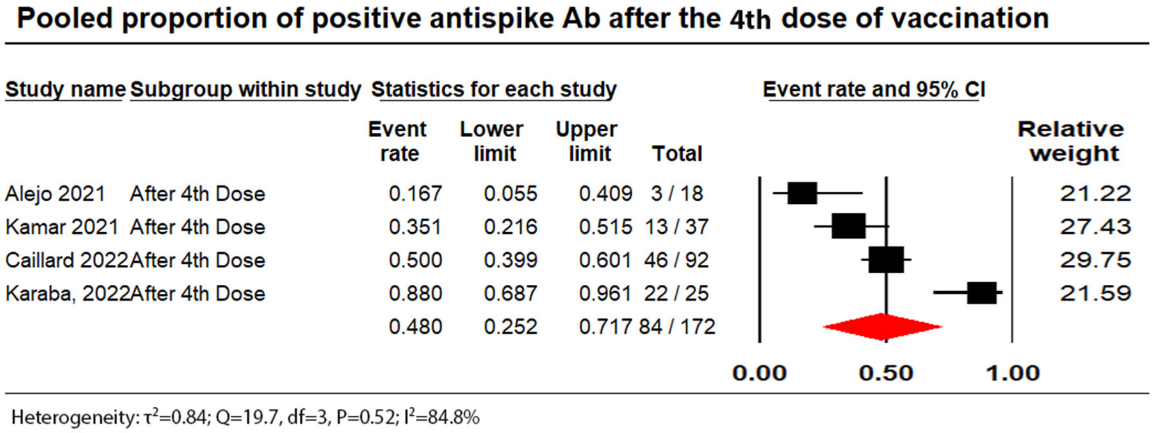

#Studies: 4 #Patients: 172 #Response: 84
#Proportion (95%CI): 0.48 (0.25 to 0.72)

The pooled proportion of positive antispike Ab after the fourth dose of vaccination from the random effects model is 0.48 (95%CI: 0.25 to 0.72; N = 172). There is a substantial heterogeneity between studies reporting seroconversion after administration of the second dose of the vaccine (I^2 = 84.8%).

Table (S4): sensitivity analysis showing the results after one study removal from the 4 studies used in the above forest plot:

Study name	Statistics with study removed			
	Point	Lower limit	Upper limit	p-Value
Alejo 2021	0.58	0.32	0.80	0.56
Kamar 2021	0.53	0.19	0.85	0.88
Caillard 2022	0.48	0.13	0.85	0.92
Karaba, 2022	0.36	0.21	0.55	0.15
Random	0.48	0.25	0.72	0.88

This table of sensitivity analysis indicates that the results are not robust since the pooled seroconversion declined to 36% when Karaba 2022 (relative weight of study is 21.2%) study removed and increased to 58% when Alejo 2021 (relative weight of study is 21.2%) study removed from the analysis. However, the low number of studies included in the analysis may explain the compromised sensitivity analysis.

Figure (S8) funnel plot of the 4 studies showing the immune response after the Fourth dose:

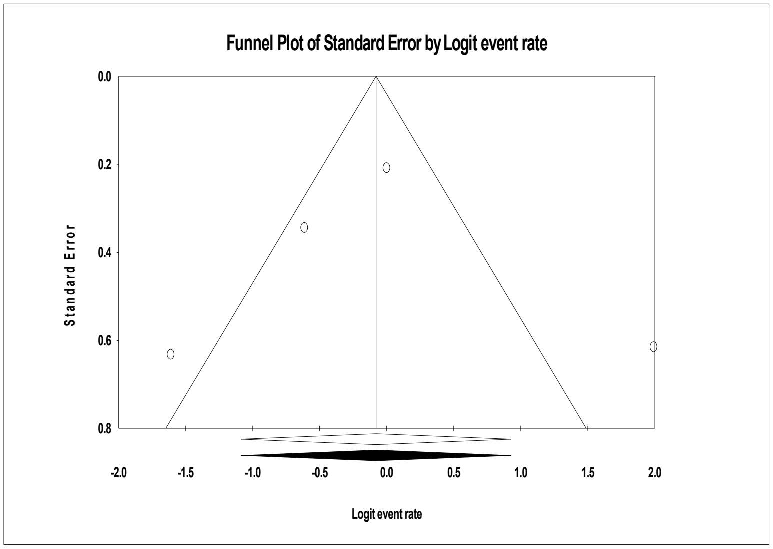

Egger's regression intercept

Intercept	0.11384
Standard error	3.62815
95% lower limit (2-tailed)	-15.49683
95% upper limit (2-tailed)	15.72450
t-value	0.03138
df	2.00000
P-value (1-tailed)	0.48891
P-value (2-tailed)	0.97782

According to the funnel plot, where the pooled studies symmetry around the mean effect size, and Egger's regression intercept test ($P > 0.05$), there is no evidence of publication bias in this analysis. Moreover, with the random effects model the point estimate and 95% confidence interval for the pooled studies using Duval and Tweedie's Trim and Fill are unchanged.

Figure (S9): Pooled proportion of +ve antispike Ab after all doses administration:

Pooled proportion of positive antispike Ab after all doses of vaccination

Study name	Subgroup within study	Event rate	Lower limit	Upper limit	Total	Relative weight
Strauss, 2021	After ALL Doses	0.807	0.739	0.861	130 / 161	2.05
Kamar, 2021	After ALL Doses	0.663	0.566	0.749	67 / 101	2.03
Hall, (A) 2021	After ALL Doses	0.550	0.424	0.670	33 / 60	1.95
Peled, 2021	After ALL Doses	0.182	0.111	0.284	14 / 77	1.88
Hall, 2021	After ALL Doses	0.314	0.238	0.402	38 / 121	2.05
Herrera, 2021	After ALL Doses	0.644	0.548	0.730	67 / 104	2.04
Boyarsky (A), 2021	After ALL Doses	0.543	0.504	0.580	357 / 658	2.18
Werbel, 2021	After ALL Doses	0.467	0.299	0.642	14 / 30	1.75
Boyarsky (B), 2021	After ALL Doses	0.586	0.550	0.621	432 / 737	2.18
Shostak, 2021	After ALL Doses	0.185	0.133	0.250	31 / 168	2.05
Rozen-Zvi , 2021	After ALL Doses	0.383	0.330	0.439	118 / 308	2.15
Ben Zadok, 2021	After ALL Doses	0.486	0.332	0.644	18 / 37	1.82
Schmidt, 2021	After ALL Doses	0.316	0.189	0.478	12 / 38	1.78
Grupper, 2021	After ALL Doses	0.375	0.298	0.459	51 / 136	2.08
Rabinowich, 2021	After ALL Doses	0.475	0.368	0.584	38 / 80	2.01
Korth, 2021	After ALL Doses	0.217	0.093	0.428	5 / 23	1.47
Chavarot, 2021	After ALL Doses	0.036	0.014	0.093	4 / 110	1.46
Benotmane, 2021	After ALL Doses	0.478	0.410	0.546	98 / 205	2.12
Marinaki, 2021	After ALL Doses	0.588	0.419	0.739	20 / 34	1.78
Boyarsky, (C), 2021	After ALL Doses	0.174	0.142	0.213	76 / 436	2.14
Bruminhent 2021	After ALL Doses	0.114	0.044	0.268	4 / 35	1.42
Alejo 2021	After ALL Doses	0.833	0.591	0.945	15 / 18	1.24
Reindl-Schwaighofer 2021	After ALL Doses	0.386	0.320	0.456	76 / 197	2.12
Peled 2021	After ALL Doses	0.667	0.567	0.754	64 / 96	2.02
Schrezenmeier 2021	After ALL Doses	0.360	0.199	0.560	9 / 25	1.64
Kamar 2021	After ALL Doses	0.486	0.332	0.644	18 / 37	1.82
Holden 2021	After ALL Doses	0.350	0.254	0.460	28 / 80	1.99
Yanis 2021	After ALL Doses	0.216	0.124	0.349	11 / 51	1.80
Sadioglu 2021	After ALL Doses	0.188	0.119	0.285	16 / 85	1.92
Benning 2022	After ALL Doses	0.304	0.232	0.386	41 / 135	2.06
Ou 2021	After ALL Doses	0.478	0.429	0.527	191 / 400	2.16
Karaba 2021	After ALL Doses	0.723	0.580	0.832	34 / 47	1.82
Kumar 2022	After ALL Doses	0.765	0.630	0.861	39 / 51	1.82
Schramm 2021	After ALL Doses	0.100	0.042	0.219	5 / 50	1.54
Caillard 2022	After ALL Doses	0.500	0.399	0.601	46 / 92	2.03
Mazzola 2021	After ALL Doses	0.286	0.215	0.368	38 / 133	2.06
Correia 2022	After ALL Doses	0.580	0.494	0.662	76 / 131	2.08
Erol 2021	After ALL Doses	0.729	0.588	0.836	35 / 48	1.83
Hamm, 2022	After ALL Doses	0.360	0.296	0.429	72 / 200	2.12
Russo, 2021	After ALL Doses	0.524	0.417	0.630	43 / 82	2.01
Debska-Slizien, 2021	After ALL Doses	0.514	0.432	0.595	73 / 142	2.09
Vaiciuniene, 2021	After ALL Doses	0.287	0.217	0.368	39 / 136	2.06
Chukwu, 2022	After ALL Doses	0.568	0.518	0.618	212 / 373	2.16
D'Offizi, 2021	After ALL Doses	0.770	0.649	0.859	47 / 61	1.87
Narasimhan, 2021	After ALL Doses	0.247	0.161	0.358	18 / 73	1.93
Davidov, 2021	After ALL Doses	0.724	0.613	0.812	55 / 76	1.95
Hod, 2021	After ALL Doses	0.433	0.348	0.523	52 / 120	2.07
Kantauskaite, 2021	After ALL Doses	0.249	0.197	0.310	56 / 225	2.11
Seija, 2022	After ALL Doses	0.282	0.232	0.337	80 / 284	2.13
Timmermann, 2021	After ALL Doses	0.780	0.696	0.845	92 / 118	2.01
Karaba, 2022	After ALL Doses	0.880	0.687	0.961	22 / 25	1.26
Saharia, 2022	After ALL Doses	0.396	0.275	0.532	21 / 53	1.91
		0.442	0.391	0.493	3251 / 7303	

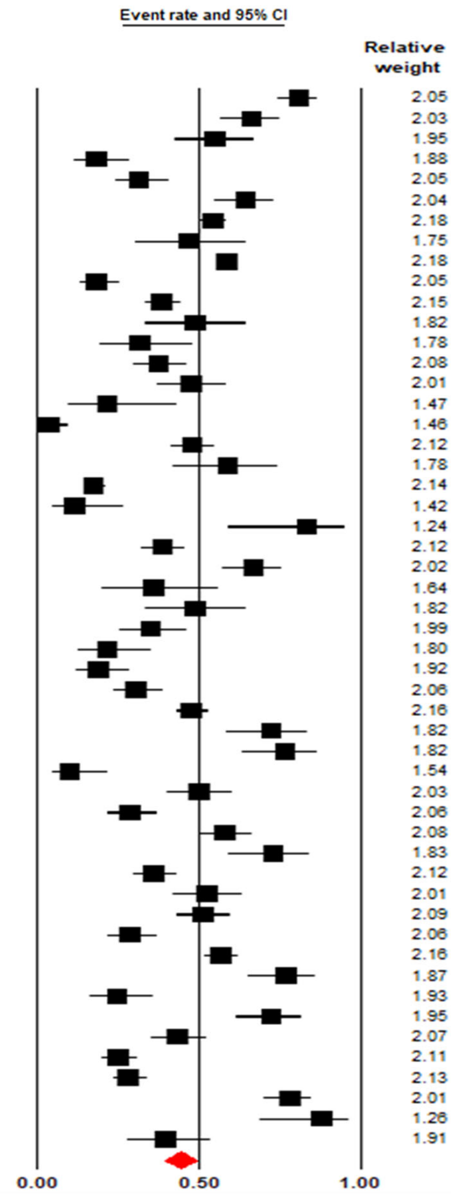

Heterogeneity: $\tau^2=0.51$; $Q=812.5$, $df=51$, $P<0.001$; $I^2=93.7\%$

#Studies: 52 #Patients: 7303 #Response: 3251
#Proportion (95%CI): 0.44 (0.39 to 0.49)

The pooled proportion of positive antispike Ab after the administration of all doses of vaccination from the random effects model is 0.44 (95%CI: 0.39 to 0.49; N = 7303). There is a considerable heterogeneity between studies reporting seroconversion after administration of all doses of the vaccine (I^2 = 93.7%).

Table (S5): sensitivity analysis showing the results after one study removal from the 52 studies used in the above forest plot:

Study name	Statistics with study removed			
	Point	Lower limit	Upper limit	p-Value
Strauss, 2021	0.43	0.38	0.48	0.01
Kamar, 2021	0.44	0.39	0.49	0.02
Hall, (A) 2021	0.44	0.39	0.49	0.02
Peled, 2021	0.45	0.40	0.50	0.05
Hall, 2021	0.44	0.39	0.50	0.04
Herrera, 2021	0.44	0.39	0.49	0.02
Boyarsky (A), 2021	0.44	0.39	0.49	0.03
Werbel, 2021	0.44	0.39	0.49	0.03
Boyarsky (B), 2021	0.44	0.39	0.49	0.02
Shostak, 2021	0.45	0.40	0.50	0.05
Rozen-Zvi , 2021	0.44	0.39	0.50	0.04
Ben Zadok, 2021	0.44	0.39	0.49	0.03
Schmidt, 2021	0.44	0.39	0.50	0.04
Grupper, 2021	0.44	0.39	0.50	0.03
Rabinowich, 2021	0.44	0.39	0.49	0.03
Korth, 2021	0.45	0.40	0.50	0.04
Chavarot, 2021	0.45	0.40	0.50	0.07
Benotmane, 2021	0.44	0.39	0.49	0.03
Marinaki, 2021	0.44	0.39	0.49	0.02
Boyarsky, (C), 2021	0.45	0.40	0.50	0.04
Bruminhent 2021	0.45	0.40	0.50	0.05
Alejo 2021	0.44	0.39	0.49	0.02
Reindl-Schwaighofer 2021	0.44	0.39	0.50	0.03
Peled 2021	0.44	0.39	0.49	0.02
Schrezenmeier 2021	0.44	0.39	0.50	0.03
Kamar 2021	0.44	0.39	0.49	0.03
Holden 2021	0.44	0.39	0.50	0.04
Yanis 2021	0.45	0.40	0.50	0.04
Sadioglu 2021	0.45	0.40	0.50	0.05
Benning 2022	0.44	0.39	0.50	0.04
Ou 2021	0.44	0.39	0.49	0.03
Karaba 2021	0.44	0.39	0.49	0.02
Kumar 2022	0.44	0.39	0.49	0.01
Schramm 2021	0.45	0.40	0.50	0.05
Caillard 2022	0.44	0.39	0.49	0.03
Mazzola 2021	0.45	0.39	0.50	0.04
Correia 2022	0.44	0.39	0.49	0.02
Erol 2021	0.44	0.39	0.49	0.02
Hamm, 2022	0.44	0.39	0.50	0.04

Russo, 2021	0.44	0.39	0.49	0.03
Debska-Slizien, 2021	0.44	0.39	0.49	0.03
Vaiciuniene, 2021	0.45	0.39	0.50	0.04
Chukwu, 2022	0.44	0.39	0.49	0.02
D’Offizi, 2021	0.43	0.39	0.49	0.01
Narasimhan, 2021	0.45	0.40	0.50	0.04
Davidov, 2021	0.44	0.39	0.49	0.02
Hod, 2021	0.44	0.39	0.49	0.03
Kantauskaite, 2021	0.45	0.40	0.50	0.04
Seija, 2022	0.45	0.39	0.50	0.04
Timmermann, 2021	0.43	0.38	0.49	0.01
Karaba, 2022	0.43	0.38	0.49	0.01
Saharia, 2022	0.44	0.39	0.50	0.03
Random	0.44	0.39	0.49	0.03

This table of sensitivity analysis indicates that the results are robust in the sense that the pooled seroconversion after the administration of all doses of vaccination will remain essentially the same (range from 43% to 45%) with any one study removed.

Figure (S10) funnel plot of the 52 studies showing the total accumulated seroconversion after the administration of the 4 doses

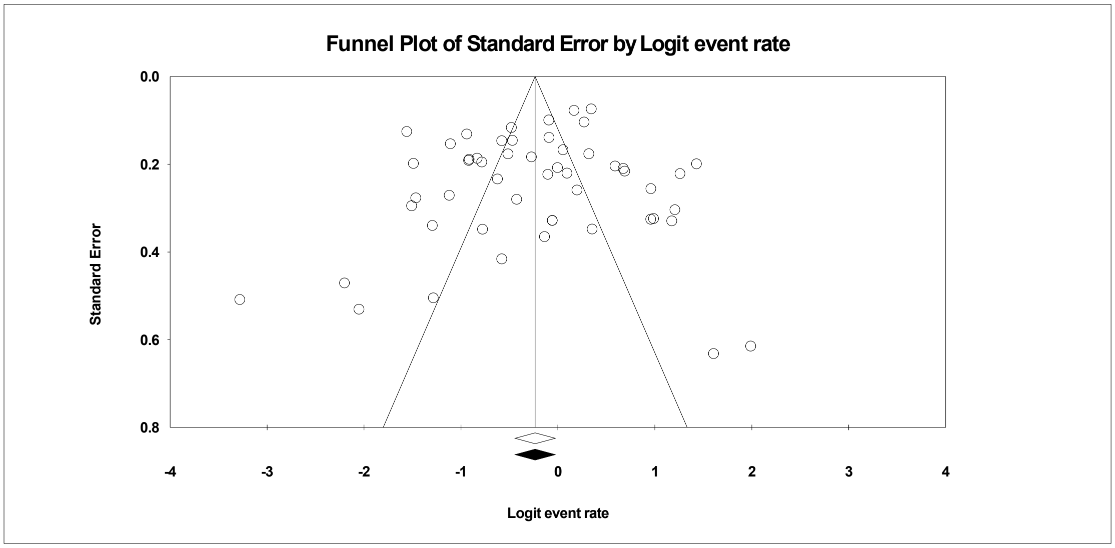

Egger's regression intercept

Intercept	-0.97796
Standard error	1.19657
95% lower limit (2-tailed)	-3.38135
95% upper limit (2-tailed)	1.42542
t-value	0.81730
df	50.00000
P-value (1-tailed)	0.20882
P-value (2-tailed)	0.41763

Duval and Tweedie's trim and fill

		Fixed Effects			Random Effects			Q Value
	Studies Trimmed	Point Estimate	Lower Limit	Upper Limit	Point Estimate	Lower Limit	Upper Limit	
**Observed values**		0.45700	0.44475	0.46931	0.44155	0.39114	0.49321	812.51658
**Adjusted values**	0	0.45700	0.44475	0.46931	0.44155	0.39114	0.49321	812.51658

According to the funnel plot, where the pooled studies symmetry around the mean effect size, and Egger's regression intercept test ($P > 0.05$), there is no evidence of publication bias in this analysis. Moreover, with the random effects model the point

estimate and 95% confidence interval for the combined studies is 0.44 (0.39, 0.49). Using Duval and Tweedie's Trim and Fill approach these values are unchanged; confirming absence of publication bias reported above from funnel plot and Egger's test.

Subgroup comaprison of seroconversion according to number of vaccine doses

Study name	Subgroup within study	Event rate	Lower limit	Upper limit	Relative weight
Strauss, 2021	After 1st Dose	0.342	0.273	0.418	6.60
Kamar, 2021	After 1st Dose	0.040	0.015	0.101	5.68
Hall, 2021	After 1st Dose	0.050	0.022	0.106	5.99
Herrera, 2021	After 1st Dose	0.260	0.184	0.352	6.50
Boyarsky (A), 2021	After 1st Dose	0.149	0.124	0.178	6.67
Boyarsky (B), 2021	After 1st Dose	0.586	0.550	0.621	6.70
Shostak, 2021	After 1st Dose	0.036	0.016	0.077	6.00
Ben Zadok, 2021	After 1st Dose	0.154	0.071	0.303	5.91
Schmidt, 2021	After 1st Dose	0.053	0.013	0.187	4.90
Chavarot, 2021	After 1st Dose	0.020	0.005	0.076	4.95
Benotmane, 2021	After 1st Dose	0.122	0.084	0.174	6.52
Boyarsky, (C), 2021	After 1st Dose	0.174	0.142	0.213	6.66
Yanis 2021	After 1st Dose	0.010	0.001	0.136	2.77
Ou 2021	After 1st Dose	0.130	0.105	0.160	6.66
Schramm 2021	After 1st Dose	0.040	0.010	0.146	4.92
Mazzola 2021	After 1st Dose	0.072	0.038	0.133	6.21
Erol 2021	After 1st Dose	0.479	0.343	0.618	6.36
		0.119	0.069	0.198	
Strauss, 2021	After 2nd Dose	0.466	0.390	0.543	4.51
Kamar, 2021	After 2nd Dose	0.364	0.275	0.463	4.14
Peled, 2021	After 2nd Dose	0.182	0.111	0.284	3.48
Hall, 2021	After 2nd Dose	0.291	0.214	0.382	4.13
Herrera, 2021	After 2nd Dose	0.385	0.296	0.481	4.20
Boyarsky (A), 2021	After 2nd Dose	0.394	0.357	0.431	4.94
Shostak, 2021	After 2nd Dose	0.181	0.125	0.254	4.05
Rozen-Zvi , 2021	After 2nd Dose	0.364	0.312	0.419	4.75
Ben Zadok, 2021	After 2nd Dose	0.357	0.204	0.546	2.78
Schmidt, 2021	After 2nd Dose	0.294	0.166	0.466	2.90
Grupper, 2021	After 2nd Dose	0.375	0.298	0.459	4.37
Rabinowich, 2021	After 2nd Dose	0.475	0.368	0.584	4.03
Korth, 2021	After 2nd Dose	0.217	0.093	0.428	2.15
Chavarot, 2021	After 2nd Dose	0.057	0.014	0.202	1.33
Benotmane, 2021	After 2nd Dose	0.361	0.298	0.429	4.59
Marinaki, 2021	After 2nd Dose	0.588	0.419	0.739	3.09
Bruminhent 2021	After 2nd Dose	0.114	0.044	0.268	2.03
Alejo 2021	After 2nd Dose	0.278	0.121	0.519	2.05
Peled 2021	After 2nd Dose	0.271	0.191	0.368	3.98
Schrezenmeier 2021	After 2nd Dose	0.019	0.001	0.244	0.43
Holden 2021	After 2nd Dose	0.350	0.254	0.460	3.95
Yanis 2021	After 2nd Dose	0.216	0.124	0.349	3.15
Sadioglu 2021	After 2nd Dose	0.188	0.119	0.285	3.61
Benning 2022	After 2nd Dose	0.304	0.232	0.386	4.30
Ou 2021	After 2nd Dose	0.478	0.429	0.527	4.85
Karaba 2021	After 2nd Dose	0.234	0.135	0.375	3.12
Schramm 2021	After 2nd Dose	0.060	0.019	0.170	1.76
Mazzola 2021	After 2nd Dose	0.218	0.156	0.296	4.13
Erol 2021	After 2nd Dose	0.250	0.148	0.390	3.20
		0.301	0.262	0.342	
Kamar, 2021	After 3rd Dose	0.263	0.185	0.358	9.45
Hall, (A) 2021	After 3rd Dose	0.433	0.315	0.560	9.09
Werbel, 2021	After 3rd Dose	0.267	0.139	0.450	7.28
Alejo 2021	After 3rd Dose	0.389	0.198	0.621	6.49
Reindl-Schwaighofer 2021	After 3rd Dose	0.386	0.320	0.456	10.24
Peled 2021	After 3rd Dose	0.396	0.303	0.497	9.66
Schrezenmeier 2021	After 3rd Dose	0.360	0.199	0.560	7.24
Kamar 2021	After 3rd Dose	0.135	0.057	0.286	6.51
Karaba 2021	After 3rd Dose	0.489	0.351	0.629	8.72
Kumar 2022	After 3rd Dose	0.765	0.630	0.861	8.27
Correia 2022	After 3rd Dose	0.580	0.494	0.662	9.97
Karaba, 2022	After 3rd Dose	0.680	0.478	0.831	7.10
		0.426	0.337	0.521	
Alejo 2021	After 4th Dose	0.167	0.055	0.409	21.22
Kamar 2021	After 4th Dose	0.351	0.216	0.515	27.43
Caillard 2022	After 4th Dose	0.500	0.399	0.601	29.75
Karaba, 2022	After 4th Dose	0.880	0.687	0.961	21.59
		0.480	0.252	0.717	
Overall		0.308	0.275	0.344	

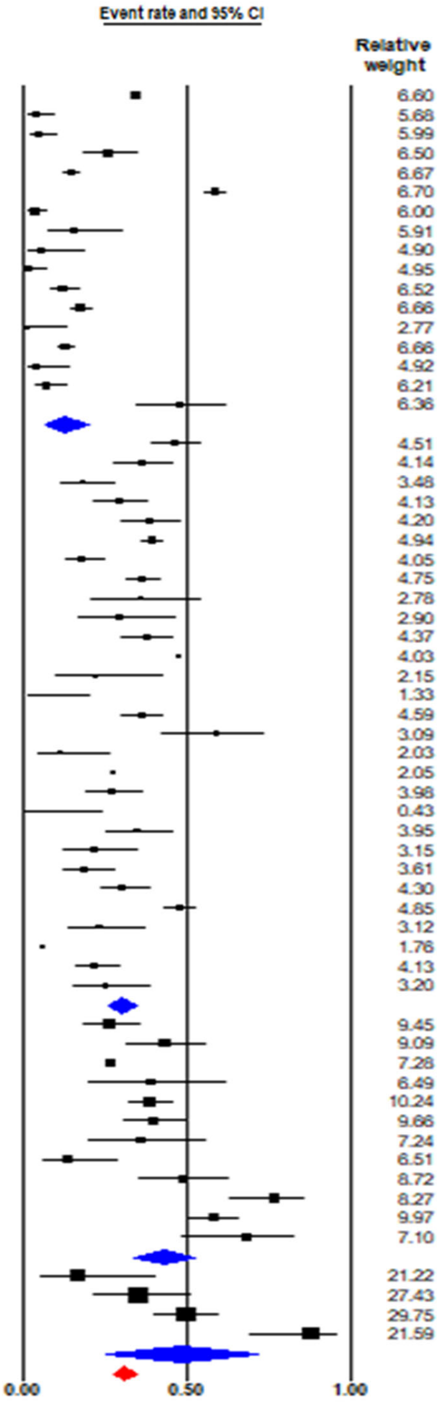

Overall heterogeneity: τ²=0.63; Q=937.3, df=61, P<0.001; I²=93.5%

#Studies: 62 (Not mutually exclusive)
#Patients: 8156 #Response: 2464
#Proportion (95%CI): 0.31 (0.28 to 0.34)

Subgroup comparison of seroconversion according to number of vaccine doses

Group by / Subgroup within study	Event rate	Lower limit	Upper limit	Total
After 1st Dose	0.119	0.069	0.198	850 / 3735
After 2nd Dose	0.301	0.262	0.342	1180 / 3433
After 3rd Dose	0.426	0.337	0.521	350 / 816
After 4th Dose	0.480	0.252	0.717	84 / 172
Overall	0.308	0.275	0.344	2464 / 8156

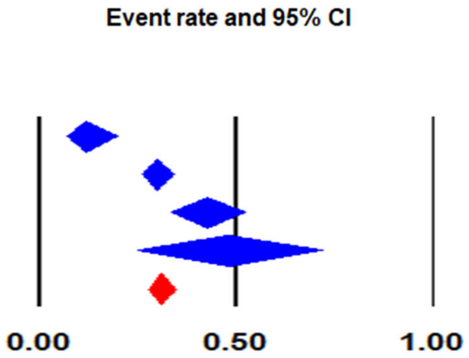

Overall heterogeneity: $\tau^2=0.63$; $Q=937.3$, $df=61$, $P<0.001$; $I^2=93.5\%$
