# Supplementary material for: Humoral and Cellular Immunity following Five Doses of COVID-19 Vaccines in Solid Organ Transplant Recipients: A Systematic Review and Meta-Analysis
Source: Vaccines (Basel). 2023 Jun 27;11(7):1166. doi: 10.3390/vaccines11071166 (PMC10384009; doi:10.3390/vaccines11071166)
Supplement: Supplementary file 1 [file vaccines-11-01166-s001.zip › File S6 Risk Factors Analysis.pdf]

Figure S1. Forest Plots of Studied Risk Factors

Age

Study name	Subgroup within study	Difference in means	Standard error	Lower limit	Upper limit	p-Value	Responders	Non-responders	Relative weight
Kamar, 2021	Age	-11.000	3.56	-17.98	-4.02	0.002	67	32	2.53
Peled, 2021	Age	-0.900	4.38	-9.49	7.69	0.837	14	63	1.85
Hall, 2021	Age	-1.837	1.78	-5.33	1.66	0.303	38	72	5.50
Boyarsky (B), 2021	Age	-1.100	8.78	-18.31	16.11	0.900	2	10	0.54
Shostak, 2021	Age	-8.100	2.95	-13.89	-2.31	0.006	31	137	3.27
Rozen-Zvi , 2021	Age	-6.020	1.61	-9.17	-2.87	0.000	112	196	5.94
Ben Zadok, 2021	Age	-18.000	5.74	-29.24	-6.76	0.002	18	19	1.18
Grupper, 2021	Age	-6.350	2.20	-10.66	-2.04	0.004	51	85	4.56
Rabinowich, 2021	Age	-5.440	2.66	-10.66	-0.22	0.041	38	42	3.71
Korth, 2021	Age	-0.900	7.01	-14.64	12.84	0.898	5	18	0.83
Benotmane, 2021	Age	-2.100	1.90	-5.82	1.62	0.268	98	106	5.23
Alejo 2021	Age	-7.200	6.18	-19.32	4.92	0.244	15	3	1.03
Reindl-Schwaighofer 2021	Age	0.000	1.78	-3.48	3.48	1.000	99	98	5.51
Peled 2021	Age	-7.000	3.03	-12.93	-1.07	0.021	64	32	3.16
Kamar 2021	Age	-2.000	22.58	-46.25	42.25	0.929	13	19	0.09
Holden 2021	Age	-7.100	3.03	-13.04	-1.16	0.019	28	52	3.16
Sadioglu 2021	Age	-10.000	3.10	-16.08	-3.92	0.001	16	69	3.06
Kumar 2022	Age	-0.300	2.08	-4.38	3.78	0.886	11	49	4.81
Caillard 2022	Age	-3.800	2.47	-8.65	1.05	0.125	46	46	4.03
Correia 2022	Age	-1.100	1.71	-4.45	2.25	0.520	76	55	5.67
Debska-Slizien, 2021	Age	-8.330	2.38	-12.99	-3.67	0.000	73	69	4.21
Vaiciuniene, 2021	Age	-1.000	2.52	-5.94	3.94	0.692	39	97	3.95
Chukwu, 2022	Age	-7.000	1.42	-9.78	-4.22	0.000	212	161	6.42
Davidov, 2021	Age	-8.340	3.98	-16.14	-0.54	0.036	55	21	2.15
Hod, 2021	Age	-2.430	2.50	-7.32	2.46	0.330	42	78	3.99
Kantauskaite, 2021	Age	-0.330	1.82	-3.89	3.23	0.856	56	169	5.41
Seija, 2022	Age	-3.670	2.92	-9.38	2.04	0.208	80	204	3.32
Timmermann, 2021	Age	-4.300	2.20	-8.61	0.01	0.051	92	26	4.56
Saharia, 2022	Age	-3.000	11.31	-25.17	19.17	0.791	10	43	0.34
		-4.364	0.67	-5.67	-3.06	0.000	1501	2071	

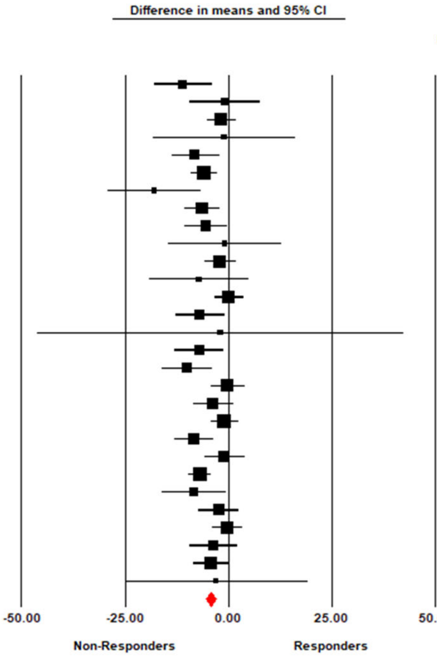

Heterogeneity: τ^2=4.94; Q=50.05, df=28, P=0.006; I^2=44%

#Studies: 29 #Patients: 3572 #Response: 1501
#Diff in mean (95%CI): -4.363 (-5.67 to -3.06) P < 0.001

The pooled mean difference of all 29 studies considering age showed that younger age significantly improves the response to vaccine (MD=-4.364, p-value<0.001). The mean age of the vaccine responders was 4.364 years younger than non-responders. Lower age is associated with higher vaccine response. The result showed moderate heterogeneity (p-value=0.006, I^2=44%). The sensitivity analysis was performed (Figure 2, below) and a random effect model was used to show the results are robust.

Antimetabolites

Study name	Subgroup within study	Odds ratio	Lower limit	Upper limit	p-Value	Responders	Non-responders	Relative weight
		Statistics for each study				Events / Total		
Strauss, 2021	Antimetabolites	0.054	0.02	0.17	0.000	12 / 55	26 / 31	2.73
Kamar, 2021	Antimetabolites	0.526	0.21	1.34	0.179	41 / 67	24 / 32	3.32
Peled, 2021	Antimetabolites	0.105	0.03	0.38	0.001	5 / 14	53 / 63	2.41
Hall, 2021	Antimetabolites	0.113	0.04	0.30	0.000	18 / 38	64 / 72	3.22
Boyarsky, 2021	Antimetabolites	0.303	0.20	0.46	0.000	167 / 259	258 / 301	5.11
Boyarsky (B), 2021	Antimetabolites	0.111	0.00	3.52	0.213	1 / 2	9 / 10	0.52
Shostak, 2021	Antimetabolites	0.258	0.08	0.81	0.020	25 / 31	129 / 137	2.75
Rozen-Zvi , 2021	Antimetabolites	0.323	0.19	0.54	0.000	66 / 112	160 / 196	4.74
Ben Zadok, 2021	Antimetabolites	0.072	0.01	0.36	0.001	5 / 18	16 / 19	1.81
Grupper, 2021	Antimetabolites	0.304	0.13	0.69	0.004	32 / 51	72 / 85	3.71
Rabinowich, 2021	Antimetabolites	0.443	0.18	1.09	0.075	15 / 38	25 / 42	3.46
Korth, 2021	Antimetabolites	0.300	0.03	2.65	0.278	3 / 5	15 / 18	1.15
Benotmane, 2021	Antimetabolites	0.412	0.20	0.83	0.013	70 / 98	91 / 106	4.11
Marinaki, 2021	Antimetabolites	0.133	0.03	0.62	0.010	5 / 20	10 / 14	1.92
Boyarsky, (C), 2021	Antimetabolites	0.136	0.08	0.23	0.000	28 / 76	292 / 360	4.69
Alejo 2021	Antimetabolites	0.771	0.03	20.02	0.876	13 / 15	3 / 3	0.58
Reindl-Schwaighofer 2021	Antimetabolites	0.384	0.07	2.03	0.259	2 / 99	5 / 98	1.73
Peled 2021	Antimetabolites	0.395	0.12	1.29	0.125	47 / 64	28 / 32	2.64
Kamar 2021	Antimetabolites	3.200	0.31	32.53	0.326	12 / 13	15 / 19	1.04
Holden 2021	Antimetabolites	0.041	0.00	0.77	0.033	23 / 28	52 / 52	0.69
Sadioglu 2021	Antimetabolites	0.686	0.23	2.07	0.503	9 / 16	45 / 69	2.85
Kumar 2022	Antimetabolites	0.568	0.14	2.28	0.425	7 / 11	37 / 49	2.19
Caillard 2022	Antimetabolites	0.758	0.27	2.13	0.600	36 / 46	38 / 46	3.04
Mazzola 2021	Antimetabolites	0.579	0.26	1.30	0.184	24 / 38	71 / 95	3.75
Correia 2022	Antimetabolites	0.234	0.10	0.53	0.001	39 / 76	45 / 55	3.70
Erol 2021	Antimetabolites	0.160	0.02	1.38	0.095	23 / 35	12 / 13	1.17
Russo, 2021	Antimetabolites	0.230	0.08	0.66	0.006	24 / 43	33 / 39	2.98
Debska-Slizien, 2021	Antimetabolites	0.371	0.16	0.88	0.025	52 / 73	60 / 69	3.56
Vaiciuniene, 2021	Antimetabolites	0.045	0.01	0.15	0.000	20 / 39	93 / 97	2.65
Chukwu, 2022	Antimetabolites	0.104	0.06	0.20	0.000	115 / 212	148 / 161	4.37
Narasimhan, 2021	Antimetabolites	1.018	0.04	26.10	0.991	18 / 18	54 / 55	0.58
Davidov, 2021	Antimetabolites	0.160	0.05	0.52	0.002	7 / 55	10 / 21	2.69
Hod, 2021	Antimetabolites	0.216	0.09	0.53	0.001	25 / 42	68 / 78	3.43
Kantauskaite, 2021	Antimetabolites	0.043	0.02	0.10	0.000	26 / 56	161 / 169	3.50
Seija, 2022	Antimetabolites	0.219	0.12	0.40	0.000	48 / 80	178 / 204	4.44
Timmermann, 2021	Antimetabolites	0.407	0.13	1.25	0.116	10 / 92	6 / 26	2.80
		0.238	0.18	0.31	0.000	1073 / 2035	2406 / 2936	

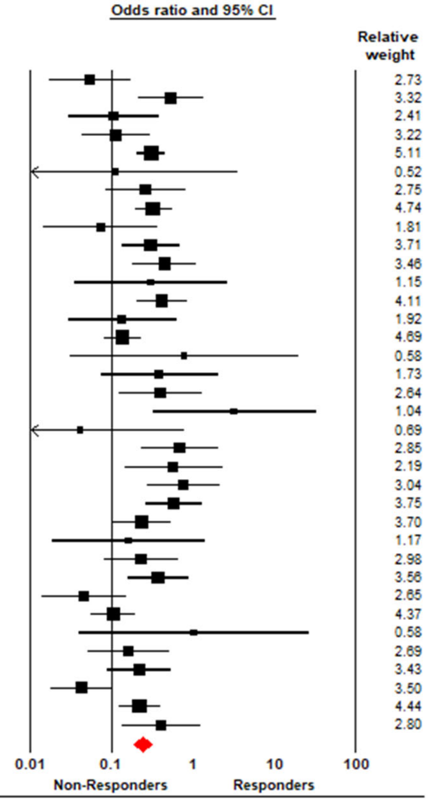

Heterogeneity: τ^2=0.30; Q=81.54, df=35, P<0.001; I^2=57%

#Studies: 36 #Patients: 4971
#Responsers: 1073/2035 #Non-responders: 2406/2936
#OR (95%CI): 0.238 (0.18 to 0.31) P < 0.001

The pooled mean difference of 36 studies considering antimetabolites showed that taking less antimetabolites significantly improves the response to vaccine (OR=0.238, p-value<0.001). Those who showed a response to the vaccine have received 76.6% less antimetabolites than who did not show a response.

The result showed moderate heterogeneity (p-value<0.001, I^2=57%). The sensitivity analysis was performed (Figure 2, below) and a random effect model was used to show the results are robust.

Antithymocyte globulin

Study name	Subgroup within study	Odds ratio	Lower limit	Upper limit	p-Value	Responders	Non-responders	Relative weight
		Statistics for each study				Events / Total		
Rozen-Zvi , 2021	Antithymocyte globulin	0.463	0.13	1.70	0.245	3 / 112	11 / 196	9.03
Grupper, 2021	Antithymocyte globulin	0.547	0.06	5.40	0.605	1 / 51	3 / 85	2.90
Benotmane, 2021	Antithymocyte globulin	0.873	0.50	1.52	0.632	55 / 98	63 / 106	49.20
Reindl-Schwaighofer 2021	Antithymocyte globulin	0.490	0.04	5.49	0.563	1 / 99	2 / 98	2.61
Sadioglu 2021	Antithymocyte globulin	1.778	0.58	5.41	0.311	7 / 16	21 / 69	12.29
Seija, 2022	Antithymocyte globulin	0.765	0.34	1.70	0.510	9 / 80	29 / 204	23.97
		0.847	0.57	1.25	0.404	76 / 456	129 / 758	

Odds ratio and 95% CI

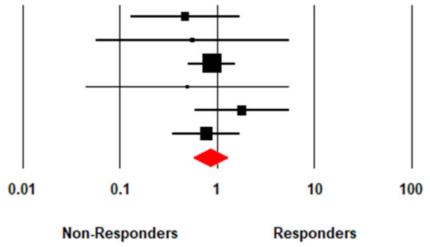

Heterogeneity: τ^2=0.0; Q=2.95, df=5, P=0.71; I^2=0%

#Studies: 6 #Patients: 1214
#Responsers: 76/456 #Non-responders: 129/758
#OR (95%CI): 0.847 (0.57 to 1.25) P = 0.404

The weighted odds ratio of all 6 studies taking antithymocyte globulin exposure into consideration is 0.847, antithymocyte globulin exposure has no significant effect on the pooled odds of vaccine immune response (pooled p-value=0.4)

The estimated heterogeneity of the model (tau2) is 0.00 and the percentage of variation across the effect sizes that is due to heterogeneity rather than chance (I2) is 0% (p-value=0.708)

Body Mass Index

Study name	Subgroup within study	Difference in means	Standard error	Lower limit	Upper limit	p-Value	Responders	Non-responders	Relative weight
		Statistics for each study					Sample size		
Peled, 2021	BMI, kg/m2	-12.000	10.54	-32.65	8.65	0.255	14	63	0.04
Rozen-Zvi , 2021	BMI, kg/m2	-0.100	0.55	-1.18	0.98	0.855	112	196	15.94
Grupper, 2021	BMI, kg/m2	-0.690	0.72	-2.11	0.73	0.341	51	85	9.14
Rabinowich, 2021	BMI, kg/m2	-0.970	1.07	-3.07	1.13	0.365	38	42	4.19
Benotmane, 2021	BMI, kg/m2	-0.030	0.67	-1.34	1.28	0.964	98	106	10.76
Peled 2021	BMI, kg/m2	-0.100	1.02	-2.10	1.90	0.922	64	32	4.61
Holden 2021	BMI, kg/m2	-1.900	1.19	-4.24	0.44	0.112	28	52	3.36
Caillard 2022	BMI, kg/m2	-0.200	1.18	-2.51	2.11	0.865	46	46	3.45
Mazzola 2021	BMI, kg/m2	-0.100	0.85	-1.77	1.57	0.906	38	95	6.64
Russo, 2021	BMI, kg/m2	-0.630	0.84	-2.28	1.02	0.454	43	39	6.79
Debska-Slizien, 2021	BMI, kg/m2	-0.080	0.71	-1.47	1.31	0.910	73	69	9.51
Chukwu, 2022	BMI, kg/m2	-0.800	0.60	-1.98	0.38	0.185	212	161	13.20
Davidov, 2021	BMI, kg/m2	-0.330	1.13	-2.55	1.89	0.771	55	21	3.74
Hod, 2021	BMI, kg/m2	-1.370	0.95	-3.23	0.49	0.150	42	78	5.30
Seija, 2022	BMI, kg/m2	-0.530	1.20	-2.89	1.83	0.660	80	204	3.31
		-0.469	0.22	-0.90	-0.04	0.032	994	1289	

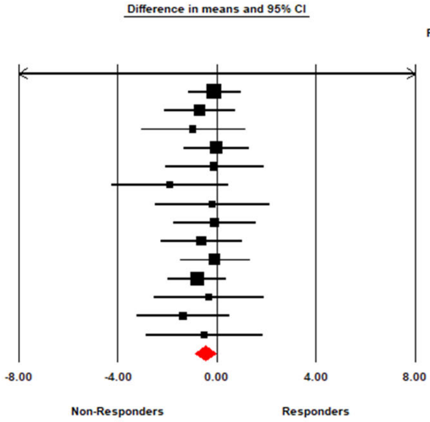

Heterogeneity: τ2=0.0; Q=5.75, df=14, P=0.97; I^2=0%

#Studies: 15 #Patients: 2283 #Response: 994
#Diff in mean (95%CI): -0.469 (-0.9 to -0.04) P = 0.032

The weighted mean difference of all 15 studies taking BMI into consideration is -0.469 with a pooled standard error of 0.219, responders have 0.469 lower pooled BMI mean difference compared to non-responders (pooled p-value=0.032). Lower BMI is associated with higher vaccine response.

The estimated heterogeneity of the model (tau^2) is 0.00 and the percentage of variation across the effect sizes that is due to heterogeneity rather than chance (I^2) is 0% (p-value=0.972)

Time from transplant

Study name	Subgroup within study	Difference in means	Standard error	Lower limit	Upper limit	p-Value	Responders	Non-responders	Relative weight
		**Statistics for each study**					**Sample size**		
Kamar, 2021	Time from transplant	0.450	1.51	-2.52	3.42	0.766	67	32	3.98
Peled, 2021	Time from transplant	1.900	2.72	-3.43	7.23	0.484	14	63	2.39
Hall, 2021	Time from transplant	0.110	0.67	-1.20	1.42	0.869	38	72	5.24
Werbel, 2021	Time from transplant	1.886	1.78	-1.61	5.38	0.290	14	40	3.56
Boyarsky (B), 2021	Time from transplant	44.370	5.88	32.85	55.89	0.000	2	10	0.76
Rozen-Zvi , 2021	Time from transplant	0.060	0.90	-1.69	1.81	0.947	112	196	4.94
Ben Zadok, 2021	Time from transplant	0.112	3.20	-6.17	6.39	0.972	18	19	1.95
Grupper, 2021	Time from transplant	2.584	1.17	0.29	4.88	0.027	51	85	4.52
Rabinowich, 2021	Time from transplant	1.450	1.29	-1.07	3.97	0.259	38	42	4.34
Korth, 2021	Time from transplant	7.900	4.47	-0.87	16.67	0.077	5	18	1.20
Benotmane, 2021	Time from transplant	1.930	1.08	-0.19	4.05	0.074	98	106	4.66
Alejo 2021	Time from transplant	7.800	4.63	-1.28	16.88	0.092	15	3	1.13
Reindl-Schwaighofer 2021	Time from transplant	0.600	0.61	-0.60	1.80	0.328	99	98	5.31
Kamar 2021	Time from transplant	6.800	2.40	2.10	11.50	0.005	13	19	2.74
Holden 2021	Time from transplant	2.100	1.99	-1.79	5.99	0.291	28	52	3.27
Sadioglu 2021	Time from transplant	1.400	1.56	-1.66	4.46	0.370	16	69	3.90
Kumar 2022	Time from transplant	2.300	1.86	-1.34	5.94	0.216	11	49	3.45
Caillard 2022	Time from transplant	1.600	1.46	-1.26	4.46	0.273	46	46	4.06
Correia 2022	Time from transplant	4.800	1.22	2.40	7.20	0.000	76	55	4.44
Debska-Slizien, 2021	Time from transplant	4.500	1.45	1.65	7.35	0.002	73	69	4.07
Vaiciuniene, 2021	Time from transplant	1.300	0.90	-0.46	3.06	0.149	39	97	4.93
Chukwu, 2022	Time from transplant	2.810	0.62	1.60	4.02	0.000	212	161	5.30
Davidov, 2021	Time from transplant	3.670	2.33	-0.90	8.24	0.115	55	21	2.82
Hod, 2021	Time from transplant	0.500	1.21	-1.87	2.87	0.680	42	78	4.46
Kantauskaite, 2021	Time from transplant	7.360	1.04	5.32	9.40	0.000	56	169	4.72
Seija, 2022	Time from transplant	1.910	1.67	-1.35	5.17	0.252	80	204	3.74
Timmermann, 2021	Time from transplant	1.900	1.42	-0.88	4.68	0.180	92	26	4.13
		2.650	0.55	1.57	3.73	0.000	1410	1899	

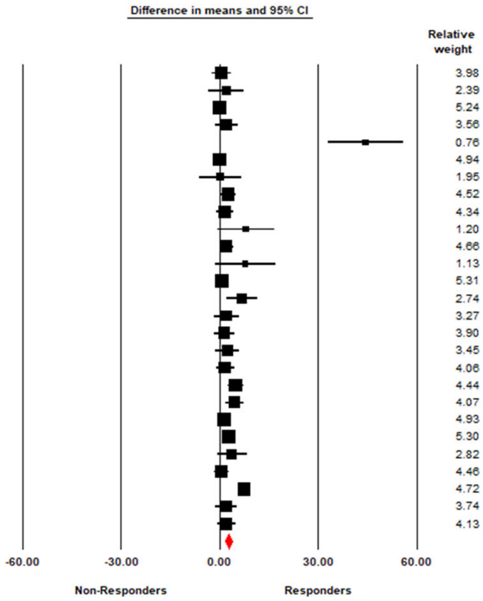

Heterogeneity: τ2=5.36; Q=118.4, df=26, P<0.001; I^2=78%

#Studies: 27 #Patients: 3309 #Response: 1410
#Diff in mean (95%CI): 2.65 (1.57 to 3.73) P < 0.001

The weighted mean difference of all 27 studies taking the time from transplant into consideration is 2.65 years with a pooled standard error of 0.551, the pooled mean difference in time from transplant shows that responders have 2.65 higher years from transplant compared to non-responders (pooled p-value<0.001) higher time from transplant is associated with higher vaccine response.

The estimated heterogeneity of the model (tau^2) is 5.355 and the percentage of variation across the effect sizes that is due to heterogeneity rather than chance (I^2) is 78% (p-value<0.001)

Calcineurin inhibitors

Study name	Subgroup within study	Statistics for each study				Events / Total		Odds ratio and 95% CI
		Odds ratio	Lower limit	Upper limit	p-Value	Responders	Non-responders	
Kamar, 2021	Calcineurin inhibitors	1.793	0.67	4.84	0.248	55 / 67	23 / 32	
Peled, 2021	Calcineurin inhibitors	2.726	0.14	52.18	0.505	14 / 14	58 / 63	
Hall, 2021	Calcineurin inhibitors	0.254	0.02	2.89	0.269	36 / 38	71 / 72	
Boyarsky, 2021	Calcineurin inhibitors	4.474	2.88	6.96	0.000	92 / 259	33 / 301	
Boyarsky (B), 2021	Calcineurin inhibitors	1.471	0.05	41.83	0.821	2 / 2	8 / 10	
Shostak, 2021	Calcineurin inhibitors	0.130	0.02	0.99	0.049	1 / 31	28 / 137	
Ben Zadok, 2021	Calcineurin inhibitors	2.857	0.48	17.11	0.250	16 / 18	14 / 19	
Grupper, 2021	Calcineurin inhibitors	0.956	0.30	3.10	0.940	46 / 51	77 / 85	
Rabinowich, 2021	Calcineurin inhibitors	0.583	0.09	3.69	0.567	35 / 38	40 / 42	
Korth, 2021	Calcineurin inhibitors	0.333	0.04	2.56	0.291	2 / 5	12 / 18	
Benotmane, 2021	Calcineurin inhibitors	0.559	0.32	0.98	0.041	48 / 98	67 / 106	
Boyarsky, (C), 2021	Calcineurin inhibitors	7.361	4.31	12.58	0.000	48 / 76	68 / 360	
Kamar 2021	Calcineurin inhibitors	0.392	0.06	2.76	0.347	10 / 13	17 / 19	
Holden 2021	Calcineurin inhibitors	6.600	0.35	123.86	0.207	28 / 28	47 / 52	
Sadioglu 2021	Calcineurin inhibitors	0.923	0.10	8.86	0.945	15 / 16	65 / 69	
Kumar 2022	Calcineurin inhibitors	0.071	0.00	1.86	0.112	10 / 11	49 / 49	
Caillard 2022	Calcineurin inhibitors	0.652	0.10	4.09	0.648	43 / 46	44 / 46	
Mazzola 2021	Calcineurin inhibitors	0.965	0.36	2.56	0.943	31 / 38	78 / 95	
Russo, 2021	Calcineurin inhibitors	1.105	0.07	18.29	0.944	42 / 43	38 / 39	
Vaiciuniene, 2021	Calcineurin inhibitors	0.428	0.13	1.37	0.152	33 / 39	90 / 97	
Chukwu, 2022	Calcineurin inhibitors	0.345	0.13	0.95	0.040	194 / 212	156 / 161	
D'Offizi, 2021	Calcineurin inhibitors	22.941	2.76	190.97	0.004	30 / 47	1 / 14	
Hod, 2021	Calcineurin inhibitors	0.985	0.34	2.88	0.978	36 / 42	67 / 78	
Kantauskaite, 2021	Calcineurin inhibitors	0.994	0.19	5.07	0.994	54 / 56	163 / 169	
Seija, 2022	Calcineurin inhibitors	0.687	0.37	1.27	0.232	60 / 80	166 / 204	
Timmermann, 2021	Calcineurin inhibitors	9.231	2.06	41.38	0.004	40 / 92	2 / 26	
		1.130	0.66	1.92	0.651	1021 / 1460	1482 / 2363	

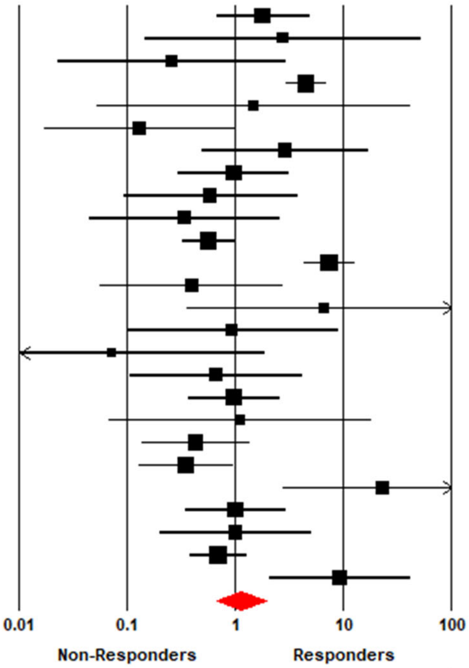

Heterogeneity: τ^2=1.2; Q=120.4, df=25, P<0.001; I^2=79.2%

#Studies: 26 #Patients: 3823
#Responsers: 1021/1460 #Non-responders: 1482/2363
#OR (95%CI): 1.13 (0.66 to 1.92) P = 0.651

The weighted odds ratio of all 26 studies taking calcineurin inhibitors use into consideration is 1.13

The calcineurin inhibitors use at the time of vaccine administration have no significant effect on the pooled odds of vaccine response (pooled p-value=0.651)

The estimated heterogeneity of the model (tau2) is 1.195 and the percentage of variation across the effect sizes that is due to heterogeneity rather than chance (I2) is 79% (p-value<0.001)

Deceased donor status

Study name	Subgroup within study	Odds ratio	Lower limit	Upper limit	p-Value	Responders	Non-responders	Relative weight
Grupper, 2021	Deceased donor status	0.468	0.22	0.99	0.047	14 / 51	38 / 85	13.70
Benotmane, 2021	Deceased donor status	1.089	0.55	2.16	0.808	79 / 98	84 / 106	14.65
Reindl-Schwaighofer 2021	Deceased donor status	1.248	0.60	2.60	0.554	83 / 99	79 / 98	13.94
Sadioglu 2021	Deceased donor status	5.521	1.74	17.55	0.004	10 / 16	16 / 69	8.76
Caillard 2022	Deceased donor status	0.617	0.20	1.90	0.400	37 / 46	40 / 46	9.05
Erol 2021	Deceased donor status	1.904	0.35	10.28	0.454	9 / 35	2 / 13	5.15
Debska-Slizien, 2021	Deceased donor status	1.348	0.35	5.24	0.667	69 / 73	64 / 69	7.09
Chukwu, 2022	Deceased donor status	0.987	0.64	1.51	0.953	135 / 212	103 / 161	18.90
Hod, 2021	Deceased donor status	0.351	0.11	1.12	0.076	4 / 42	18 / 78	8.75
		1.005	0.65	1.55	0.980	440 / 672	444 / 725	

Odds ratio and 95% CI

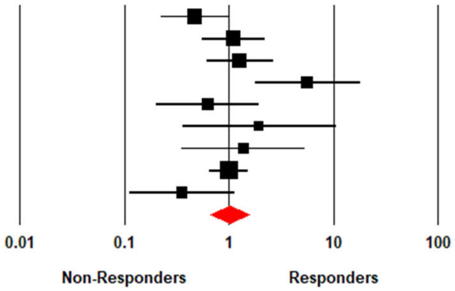

Heterogeneity: $\tau^2=0.21$; Q=17.32, df=8, P=0.027; $I^2=53.8\%$

#Studies: 9 #Patients: 1397
#Responsers: 440/672 #Non-responders: 444/725
#OR (95%CI): 1.005 (0.65 to 1.55) P = 0.98

The weighted odds ratio of all 9 studies taking decreased donor status into consideration is 1.005

Decreased donor status has no significant effect on the pooled odds of vaccine response (pooled p-value=0.98)

The estimated heterogeneity of the model (tau2) is 0.212 and the percentage of variation across the effect sizes that is due to heterogeneity rather than chance (I2) is 54% (p-value=0.027)

Gender (Males)

Study name	Subgroup within study	Odds ratio	Lower limit	Upper limit	p-Value	Responders	Non-responders	Relative weight
Strauss, 2021	Gender (males)	0.873	0.36	2.12	0.764	23 / 55	14 / 31	1.98
Kamar, 2021	Gender (males)	0.857	0.34	2.17	0.745	46 / 67	23 / 32	1.81
Peled, 2021	Gender (males)	0.966	0.29	3.24	0.955	9 / 14	41 / 63	1.06
Hall, 2021	Gender (males)	0.706	0.31	1.62	0.412	24 / 38	51 / 72	2.25
Boyarsky, 2021	Gender (males)	0.942	0.67	1.32	0.732	103 / 259	124 / 301	13.58
Werbel, 2021	Gender (males)	1.125	0.33	3.86	0.851	6 / 14	16 / 40	1.02
Boyarsky (B), 2021	Gender (males)	0.200	0.01	5.20	0.333	0 / 2	5 / 10	0.15
Shostak, 2021	Gender (males)	1.062	0.46	2.44	0.888	21 / 31	91 / 137	2.25
Rozen-Zvi , 2021	Gender (males)	1.309	0.80	2.14	0.282	76 / 112	121 / 196	6.48
Ben Zadok, 2021	Gender (males)	0.412	0.07	2.59	0.344	14 / 18	17 / 19	0.46
Grupper, 2021	Gender (males)	1.208	0.58	2.50	0.612	34 / 51	53 / 85	2.93
Rabinowich, 2021	Gender (males)	1.400	0.53	3.68	0.495	28 / 38	28 / 42	1.67
Korth, 2021	Gender (males)	0.667	0.09	4.99	0.693	2 / 5	9 / 18	0.38
Benotmane, 2021	Gender (males)	1.141	0.64	2.02	0.652	64 / 98	66 / 106	4.76
Marinaki, 2021	Gender (males)	1.091	0.20	5.87	0.919	16 / 20	11 / 14	0.55
Boyarsky, (C), 2021	Gender (males)	0.864	0.51	1.45	0.581	27 / 75	138 / 350	5.80
Alejo 2021	Gender (males)	2.286	0.17	30.96	0.534	8 / 15	1 / 3	0.23
Reindl-Schwaighofer 2021	Gender (males)	0.936	0.53	1.65	0.819	57 / 99	58 / 98	4.85
Peled 2021	Gender (males)	2.235	0.90	5.57	0.084	49 / 64	19 / 32	1.87
Kamar 2021	Gender (males)	0.500	0.12	2.10	0.344	6 / 13	12 / 19	0.76
Holden 2021	Gender (males)	0.733	0.29	1.85	0.510	14 / 28	30 / 52	1.83
Sadioglu 2021	Gender (males)	0.343	0.10	1.17	0.087	4 / 16	34 / 69	1.04
Kumar 2022	Gender (males)	1.108	0.29	4.30	0.882	7 / 11	30 / 49	0.85
Caillard 2022	Gender (males)	1.865	0.76	4.61	0.177	35 / 46	29 / 46	1.90
Mazzola 2021	Gender (males)	1.356	0.59	3.14	0.477	28 / 38	64 / 95	2.21
Correia 2022	Gender (males)	1.260	0.61	2.60	0.532	51 / 76	34 / 55	2.97
Erol 2021	Gender (males)	1.284	0.32	5.21	0.727	26 / 35	9 / 13	0.79
Russo, 2021	Gender (males)	0.879	0.37	2.11	0.773	24 / 43	23 / 39	2.02
Debska-Slizien, 2021	Gender (males)	1.167	0.60	2.28	0.650	44 / 73	39 / 69	3.49
Vaiciuniene, 2021	Gender (males)	0.987	0.46	2.12	0.973	24 / 39	60 / 97	2.66
Chukwu, 2022	Gender (males)	1.342	0.88	2.04	0.170	136 / 212	92 / 161	8.83
Narasimhan, 2021	Gender (males)	0.829	0.28	2.49	0.739	11 / 18	36 / 55	1.29
Davidov, 2021	Gender (males)	1.650	0.60	4.54	0.332	33 / 55	10 / 21	1.52
Hod, 2021	Gender (males)	0.700	0.28	1.75	0.445	32 / 42	64 / 78	1.86
Kantauskaite, 2021	Gender (males)	0.826	0.44	1.55	0.551	35 / 56	113 / 169	3.94
Seija, 2022	Gender (males)	0.966	0.57	1.63	0.898	46 / 80	119 / 204	5.68
Timmermann, 2021	Gender (males)	0.446	0.16	1.22	0.115	55 / 92	20 / 26	1.55
Saharia, 2022	Gender (males)	0.303	0.07	1.26	0.101	5 / 10	33 / 43	0.76
		1.016	0.90	1.15	0.806	1223 / 2058	1737 / 3009	

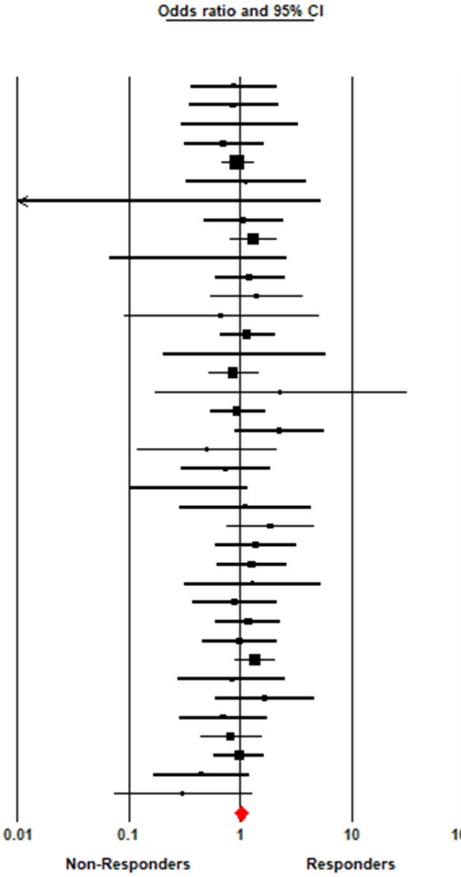

Heterogeneity: τ^2=0.0; Q=25.28, df=37, P=0.928; I^2=0%

#Studies: 38 #Patients: 5067
#Responsers: 1223/2058 #Non-responders: 1737/3009
#OR (95%CI): 1.016 (0.9 to 1.15) P = 0.806

The weighted odds ratio of all studies taking gender into consideration is 1.016

Gender has no significant effect on the pooled odds of vaccine response (pooled p-value=0.8)

The estimated heterogeneity of the model (tau^2) is 0.000 and the percentage of variation across the effect sizes that is due to heterogeneity rather than chance (I^2) is 0% (p-value=0.928)

mTOR inhibitors

Study name	Subgroup within study	Statistics for each study: Odds ratio	Lower limit	Upper limit	p-Value	Events / Total: Responders	Non-responders	Relative weight
Kamar, 2021	mTOR inhibitors	1.746	0.65	4.66	0.265	22 / 67	7 / 32	5.67
Peled, 2021	mTOR inhibitors	8.509	2.38	30.36	0.001	9 / 14	11 / 63	4.27
Hall, 2021	mTOR inhibitors	1.294	0.34	4.90	0.704	4 / 38	6 / 72	4.04
Boyarsky, 2021	mTOR inhibitors	4.474	2.88	6.96	0.000	92 / 259	33 / 301	9.18
Boyarsky (B), 2021	mTOR inhibitors	0.680	0.02	19.34	0.821	0 / 2	2 / 10	0.93
Shostak, 2021	mTOR inhibitors	0.130	0.02	0.99	0.049	1 / 31	28 / 137	2.19
Rozen-Zvi , 2021	mTOR inhibitors	1.103	0.48	2.52	0.816	10 / 112	16 / 196	6.58
Ben Zadok, 2021	mTOR inhibitors	4.457	1.11	17.90	0.035	13 / 18	7 / 19	3.82
Grupper, 2021	mTOR inhibitors	1.739	0.48	6.33	0.401	5 / 51	5 / 85	4.19
Rabinowich, 2021	mTOR inhibitors	1.494	0.54	4.13	0.439	11 / 38	9 / 42	5.47
Korth, 2021	mTOR inhibitors	0.875	0.07	10.21	0.915	1 / 5	4 / 18	1.61
Benotmane, 2021	mTOR inhibitors	2.425	1.03	5.69	0.042	18 / 98	9 / 106	6.42
Peled 2021	mTOR inhibitors	3.783	1.02	13.99	0.046	18 / 64	3 / 32	4.13
Kamar 2021	mTOR inhibitors	1.667	0.33	8.37	0.535	4 / 13	4 / 19	3.12
Holden 2021	mTOR inhibitors	1.889	0.11	31.40	0.657	1 / 28	1 / 52	1.27
Sadioglu 2021	mTOR inhibitors	1.384	0.05	35.53	0.844	0 / 16	1 / 69	0.98
Kumar 2022	mTOR inhibitors	0.880	0.09	8.38	0.911	1 / 11	5 / 49	1.86
Caillard 2022	mTOR inhibitors	1.355	0.46	4.01	0.583	9 / 46	7 / 46	5.12
Mazzola 2021	mTOR inhibitors	1.141	0.45	2.90	0.782	8 / 38	18 / 95	5.94
Russo, 2021	mTOR inhibitors	1.419	0.37	5.46	0.611	6 / 43	4 / 39	3.98
Davidov, 2021	mTOR inhibitors	0.392	0.11	1.46	0.162	6 / 55	5 / 21	4.10
Hod, 2021	mTOR inhibitors	1.423	0.30	6.68	0.655	3 / 42	4 / 78	3.31
Kantauskaite, 2021	mTOR inhibitors	4.256	0.92	19.64	0.063	4 / 56	3 / 169	3.36
Seija, 2022	mTOR inhibitors	3.920	1.97	7.81	0.000	22 / 80	18 / 204	7.49
Timmermann, 2021	mTOR inhibitors	0.869	0.03	21.96	0.932	1 / 92	0 / 26	0.99
		1.870	1.33	2.62	0.000	269 / 1317	210 / 1980	

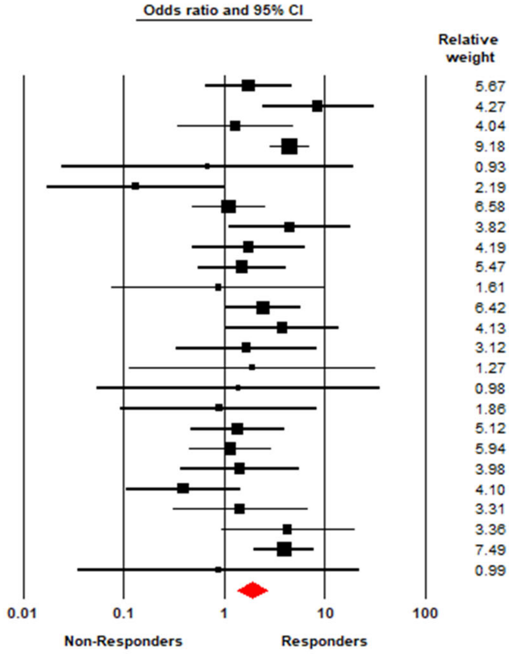

Heterogeneity: $\tau^2=0.27$; Q=43.04, df=24, P=0.010; $I^2=44.2\%$

#Studies: 25 #Patients: 3297
#Responsers: 269/1317 #Non-responders: 210/1980
#OR (95%CI): 1.87 (1.33 to 2.62) P < 0.001

The weighted odds ratio of all studies taking mTOR inhibitors into consideration is 1.87

mTOR inhibitors significantly increase the pooled odds of vaccine response by 87% (pooled p-value<0.001) Higher mTOR inhibitors is associated with higher vaccine response

The estimated heterogeneity of the model (tau^2) is 0.27 and the percentage of variation across the effect sizes that is due to heterogeneity rather than chance (I^2) is 44% (p-value=0.01)

Rituximab

Study name	Subgroup within study	Odds ratio	Lower limit	Upper limit	p-Value	Responders	Non-responders	Relative weight
		Statistics for each study				Events / Total		
Grupper, 2021	Rituximab	0.547	0.06	5.40	0.605	1 / 51	3 / 85	44.99
Rozen-Zvi , 2021	Rituximab	0.130	0.01	2.33	0.166	0 / 112	6 / 196	28.34
Seija, 2022	Rituximab	0.358	0.02	7.00	0.498	0 / 80	3 / 204	26.67
		0.325	0.07	1.51	0.152	1 / 243	12 / 485	

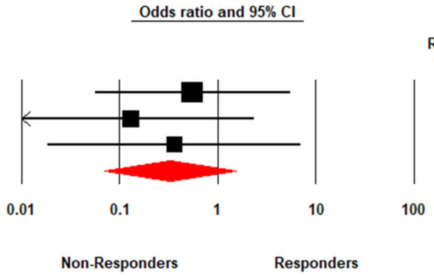

Heterogeneity: τ^2=0; Q=0.588, df=2, P=0.745; I^2=0%

#Studies: 3 #Patients: 728
#Responsers: 1/243 #Non-responders: 12/485
#OR (95%CI): 0.325 (0.07 to 1.51) P = 0.152

The weighted odds ratio of all 3 studies taking Rituximab exposure into consideration is 0.325

Rituximab exposure has on significant effect on the pooled odds of vaccine response (pooled p-value=0.152)

The estimated heterogeneity of the model (tau2) is 0 and the percentage of variation across the effect sizes that is due to heterogeneity rather than chance (I2) is 0% (p-value=0.745)

Lymphocyte count

Study name	Subgroup within study	Difference in means	Standard error	Lower limit	Upper limit	p-Value	Responders	Non-responders	Relative weight
		Statistics for each study					Sample size		
Grupper, 2021	Lymphocyte count	0.440	0.15	0.14	0.74	0.004	51	85	27.70
Hod, 2021	Lymphocyte count	0.280	0.28	-0.27	0.83	0.314	42	78	8.39
Kumar 2022	Lymphocyte count	0.150	0.16	-0.17	0.47	0.353	11	49	24.86
Rabinowich, 2021	Lymphocyte count	0.165	0.17	-0.17	0.50	0.328	38	42	22.76
Seija, 2022	Lymphocyte count	0.388	0.23	-0.07	0.84	0.093	80	204	12.13
Vaiciuniene, 2021	Lymphocyte count	0.500	0.39	-0.27	1.27	0.205	39	97	4.16
		0.288	0.08	0.13	0.45	0.000	261	555	

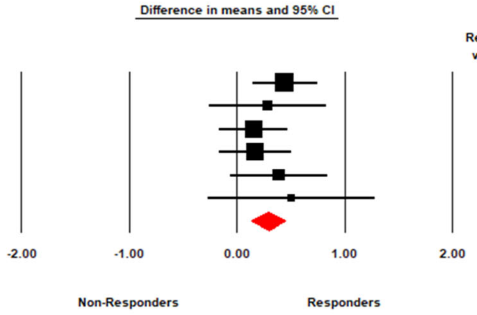

Heterogeneity: τ^2=0; Q=2.72, df=5, P=0.743; I^2=0%

#Studies: 6 #Patients: 816 #Responsers: 261
#Diff mean (95%CI): 0.288 (0.13 to 0.45) P < 0.001

The weighted mean difference of all 6 studies taking lymphocyte count into consideration is 0.288 with a pooled standard error of 0.081, the pooled mean difference in lymphocyte count shows that responders have increased lymphocyte count by 0.288 units compared to non-responders(pooled p-value<0.001). Higher lymphocyte count is associated with higher vaccine response.

The estimated heterogeneity of the model (tau2) is 0.00 and the percentage of variation across the effect sizes that is due to heterogeneity rather than chance (I2) is 0% (p-value=0.743)

Figure S2: Sensitivity Analysis

Age

Age sensitivity analysis

Study name	Subgroup within study	Point	Standard error	Variance	Lower limit	Upper limit	Z-Value	p-Value
Kamar, 2021	Age	-4.172	0.657	0.432	-5.460	-2.883	-6.346	0.000
Peled, 2021	Age	-4.437	0.680	0.462	-5.769	-3.105	-6.528	0.000
Hall, 2021	Age	-4.519	0.694	0.481	-5.878	-3.159	-6.515	0.000
Boyarsky (B), 2021	Age	-4.391	0.677	0.458	-5.717	-3.065	-6.489	0.000
Shostak, 2021	Age	-4.235	0.677	0.458	-5.561	-2.909	-6.260	0.000
Rozen-Zvi , 2021	Age	-4.274	0.699	0.488	-5.643	-2.904	-6.116	0.000
Ben Zadok, 2021	Age	-4.167	0.635	0.403	-5.410	-2.923	-6.566	0.000
Grupper, 2021	Age	-4.278	0.691	0.477	-5.632	-2.924	-6.193	0.000
Rabinowich, 2021	Age	-4.336	0.692	0.478	-5.692	-2.981	-6.269	0.000
Korth, 2021	Age	-4.402	0.677	0.459	-5.729	-3.074	-6.499	0.000
Benotmane, 2021	Age	-4.500	0.696	0.484	-5.864	-3.137	-6.470	0.000
Alejo 2021	Age	-4.342	0.678	0.460	-5.671	-3.014	-6.406	0.000
Reindl-Schwaighofer 2021	Age	-4.590	0.661	0.438	-5.886	-3.293	-6.939	0.000
Peled 2021	Age	-4.283	0.683	0.467	-5.623	-2.944	-6.269	0.000
Kamar 2021	Age	-4.375	0.675	0.456	-5.699	-3.051	-6.477	0.000
Holden 2021	Age	-4.280	0.683	0.466	-5.618	-2.942	-6.268	0.000
Sadioglu 2021	Age	-4.167	0.661	0.437	-5.463	-2.871	-6.303	0.000
Kumar 2022	Age	-4.556	0.674	0.454	-5.877	-3.235	-6.760	0.000
Caillard 2022	Age	-4.405	0.696	0.484	-5.769	-3.042	-6.332	0.000
Correia 2022	Age	-4.555	0.683	0.466	-5.893	-3.217	-6.672	0.000
Debska-Slizien, 2021	Age	-4.176	0.671	0.450	-5.491	-2.860	-6.223	0.000
Vaiciuniene, 2021	Age	-4.505	0.683	0.467	-5.844	-3.166	-6.592	0.000
Chukwu, 2022	Age	-4.164	0.679	0.460	-5.494	-2.834	-6.137	0.000
Davidov, 2021	Age	-4.278	0.676	0.457	-5.604	-2.953	-6.325	0.000
Hod, 2021	Age	-4.458	0.692	0.479	-5.814	-3.101	-6.441	0.000
Kantauskaite, 2021	Age	-4.576	0.670	0.449	-5.889	-3.263	-6.831	0.000
Seija, 2022	Age	-4.403	0.691	0.478	-5.758	-3.048	-6.369	0.000
Timmermann, 2021	Age	-4.387	0.699	0.489	-5.757	-3.016	-6.273	0.000
Saharia, 2022	Age	-4.378	0.677	0.458	-5.704	-3.052	-6.471	0.000
		-4.364	0.668	0.446	-5.673	-3.055	-6.535	0.000

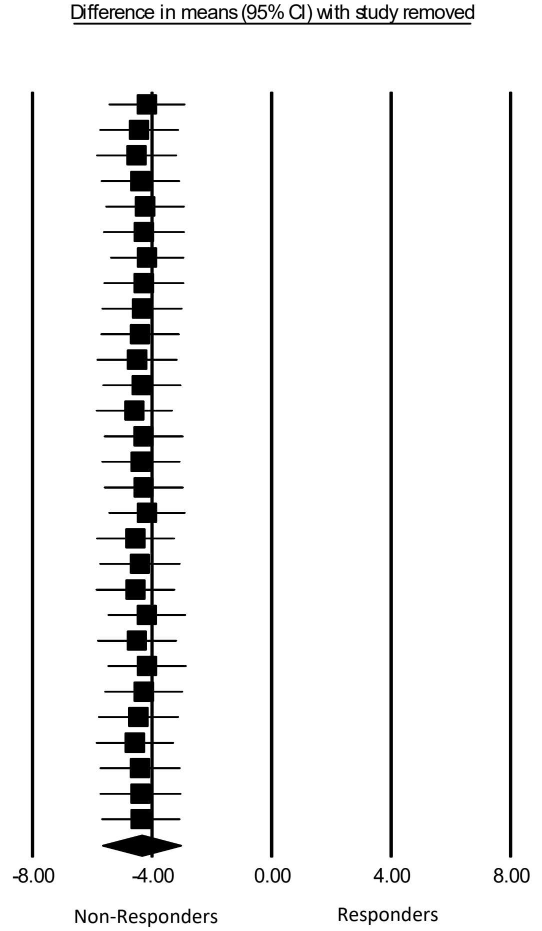

After removing one study, the p- value did not lose the significance of the point estimates and this means that there is a high heterogeneity among the studies, and this may lead to a decreased certainty of the results due to wide variability between studies.

Antimetabolites

Antimetabolites sensitivity

Study name	Subgroup within study	Statistics with study removed				
		Point	Lower limit	Upper limit	Z-Value	p-Value
Strauss, 2021	Antimetabolites	0.248	0.192	0.320	-10.676	0.000
Kamar, 2021	Antimetabolites	0.232	0.178	0.302	-10.858	0.000
Peled, 2021	Antimetabolites	0.243	0.187	0.316	-10.519	0.000
Hall, 2021	Antimetabolites	0.244	0.187	0.318	-10.456	0.000
Boyarsky, 2021	Antimetabolites	0.235	0.178	0.310	-10.225	0.000
Boyarsky (B), 2021	Antimetabolites	0.239	0.184	0.311	-10.690	0.000
Shostak, 2021	Antimetabolites	0.237	0.182	0.310	-10.550	0.000
Rozen-Zvi , 2021	Antimetabolites	0.234	0.178	0.308	-10.406	0.000
Ben Zadok, 2021	Antimetabolites	0.243	0.187	0.316	-10.593	0.000
Grupper, 2021	Antimetabolites	0.236	0.180	0.309	-10.515	0.000
Rabinowich, 2021	Antimetabolites	0.233	0.178	0.304	-10.743	0.000
Korth, 2021	Antimetabolites	0.237	0.182	0.309	-10.686	0.000
Benotmane, 2021	Antimetabolites	0.232	0.178	0.304	-10.692	0.000
Marinaki, 2021	Antimetabolites	0.241	0.185	0.314	-10.557	0.000
Boyarsky, (C), 2021	Antimetabolites	0.245	0.187	0.320	-10.334	0.000
Alejo 2021	Antimetabolites	0.236	0.182	0.307	-10.788	0.000
Reindl-Schwaighofer 2021	Antimetabolites	0.236	0.181	0.307	-10.703	0.000
Peled 2021	Antimetabolites	0.235	0.180	0.306	-10.689	0.000
Kamar 2021	Antimetabolites	0.232	0.179	0.299	-11.204	0.000
Holden 2021	Antimetabolites	0.241	0.186	0.313	-10.696	0.000
Sadioglu 2021	Antimetabolites	0.231	0.178	0.300	-10.992	0.000
Kumar 2022	Antimetabolites	0.233	0.179	0.304	-10.826	0.000
Caillard 2022	Antimetabolites	0.230	0.177	0.298	-11.106	0.000
Mazzola 2021	Antimetabolites	0.230	0.177	0.299	-11.000	0.000
Correia 2022	Antimetabolites	0.238	0.182	0.312	-10.421	0.000
Erol 2021	Antimetabolites	0.239	0.184	0.311	-10.636	0.000
Russo, 2021	Antimetabolites	0.238	0.182	0.311	-10.503	0.000
Debska-Slizien, 2021	Antimetabolites	0.234	0.179	0.306	-10.631	0.000
Vaiciuniene, 2021	Antimetabolites	0.249	0.193	0.321	-10.744	0.000
Chukwu, 2022	Antimetabolites	0.247	0.191	0.321	-10.513	0.000
Narasimhan, 2021	Antimetabolites	0.236	0.182	0.307	-10.818	0.000
Davidov, 2021	Antimetabolites	0.241	0.184	0.314	-10.488	0.000
Hod, 2021	Antimetabolites	0.239	0.183	0.312	-10.439	0.000
Kantauskaite, 2021	Antimetabolites	0.253	0.199	0.323	-11.085	0.000
Seija, 2022	Antimetabolites	0.239	0.182	0.314	-10.275	0.000
Timmermann, 2021	Antimetabolites	0.234	0.180	0.306	-10.698	0.000
		0.238	0.183	0.309	-10.809	0.000

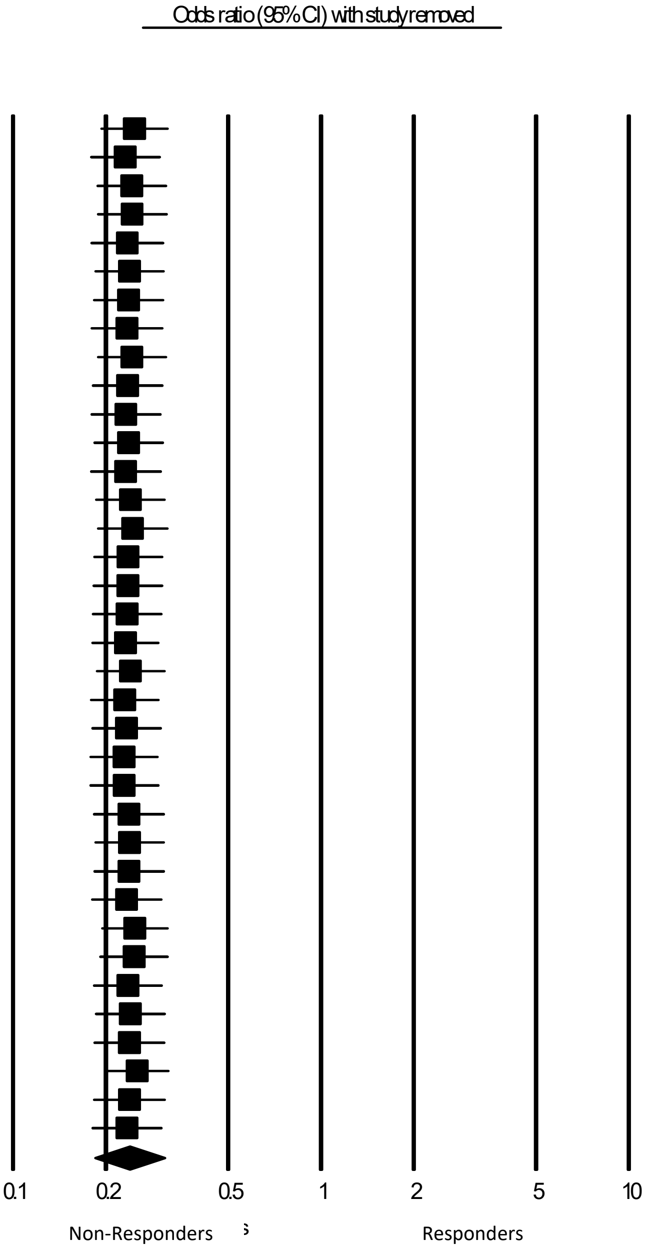

After removing one study, the p- value did not lose the significance of the point estimates and this means that there is a high heterogeneity among the studies and this may lead to a decreased certainty of the results due to wide variability between studies.

Time from transplant

Time from transplant sensitivity

Study name	Subgroup within study	Point	Standard error	Variance	Lower limit	Upper limit	Z-Value	p-Value
		Statistics with study removed						
Kamar, 2021	Time from transplant	2.750	0.568	0.323	1.637	3.864	4.840	0.000
Peled, 2021	Time from transplant	2.675	0.562	0.316	1.573	3.777	4.759	0.000
Hall, 2021	Time from transplant	2.804	0.575	0.331	1.676	3.931	4.873	0.000
Werbel, 2021	Time from transplant	2.688	0.568	0.322	1.576	3.801	4.735	0.000
Boyarsky (B), 2021	Time from transplant	2.238	0.422	0.178	1.410	3.066	5.298	0.000
Rozen-Zvi , 2021	Time from transplant	2.795	0.572	0.328	1.673	3.917	4.884	0.000
Ben Zadok, 2021	Time from transplant	2.705	0.560	0.313	1.609	3.802	4.835	0.000
Grupper, 2021	Time from transplant	2.671	0.575	0.331	1.543	3.798	4.643	0.000
Rabinowich, 2021	Time from transplant	2.720	0.573	0.329	1.596	3.844	4.743	0.000
Korth, 2021	Time from transplant	2.585	0.554	0.307	1.500	3.671	4.670	0.000
Benotmane, 2021	Time from transplant	2.706	0.578	0.334	1.573	3.839	4.682	0.000
Alejo 2021	Time from transplant	2.590	0.554	0.307	1.505	3.676	4.677	0.000
Reindl-Schwaighofer 2021	Time from transplant	2.796	0.587	0.345	1.644	3.947	4.759	0.000
Kamar 2021	Time from transplant	2.527	0.554	0.307	1.441	3.614	4.559	0.000
Holden 2021	Time from transplant	2.678	0.566	0.320	1.568	3.787	4.730	0.000
Sadioglu 2021	Time from transplant	2.712	0.570	0.325	1.596	3.829	4.761	0.000
Kumar 2022	Time from transplant	2.672	0.567	0.322	1.561	3.783	4.712	0.000
Caillard 2022	Time from transplant	2.707	0.571	0.326	1.588	3.827	4.741	0.000
Correia 2022	Time from transplant	2.548	0.562	0.316	1.446	3.649	4.532	0.000
Debska-Slizien, 2021	Time from transplant	2.574	0.564	0.318	1.468	3.680	4.562	0.000
Vaiciuniene, 2021	Time from transplant	2.746	0.582	0.339	1.605	3.887	4.717	0.000
Chukwu, 2022	Time from transplant	2.688	0.596	0.355	1.520	3.856	4.509	0.000
Davidov, 2021	Time from transplant	2.626	0.563	0.317	1.523	3.729	4.666	0.000
Hod, 2021	Time from transplant	2.762	0.572	0.327	1.642	3.882	4.833	0.000
Kantauskaite, 2021	Time from transplant	2.321	0.510	0.260	1.322	3.321	4.553	0.000
Seija, 2022	Time from transplant	2.690	0.569	0.324	1.575	3.805	4.729	0.000
Timmermann, 2021	Time from transplant	2.696	0.572	0.327	1.576	3.817	4.716	0.000
		2.650	0.551	0.304	1.570	3.731	4.807	0.000

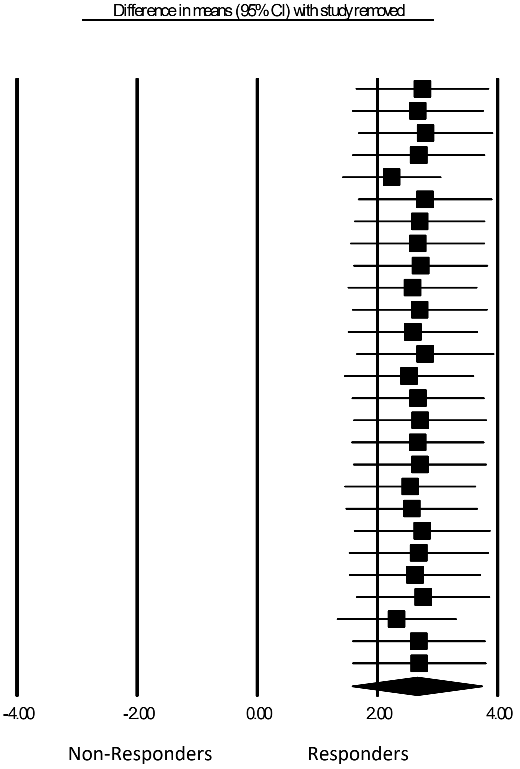

After removing one study, the p- value did not lose the significance of the point estimates and this means that there is a high heterogeneity among the studies and this may lead to a decreased certainty of the results due to wide variability between studies.

Calcineurin

Calcineurin inhibitors sensitivity

Study name	Subgroup within study	Statistics with study removed				
		Point	Lower limit	Upper limit	Z-Value	p-Value
Kamar, 2021	Calcineurin inhibitors	1.100	0.630	1.921	0.336	0.737
Peled, 2021	Calcineurin inhibitors	1.108	0.646	1.901	0.373	0.709
Hall, 2021	Calcineurin inhibitors	1.178	0.688	2.017	0.597	0.550
Boyarsky, 2021	Calcineurin inhibitors	1.038	0.608	1.773	0.137	0.891
Boyarsky (B), 2021	Calcineurin inhibitors	1.124	0.656	1.927	0.426	0.670
Shostak, 2021	Calcineurin inhibitors	1.216	0.714	2.071	0.721	0.471
Ben Zadok, 2021	Calcineurin inhibitors	1.091	0.632	1.881	0.312	0.755
Grupper, 2021	Calcineurin inhibitors	1.138	0.655	1.976	0.459	0.646
Rabinowich, 2021	Calcineurin inhibitors	1.157	0.672	1.993	0.528	0.598
Korth, 2021	Calcineurin inhibitors	1.177	0.686	2.019	0.593	0.553
Benotmane, 2021	Calcineurin inhibitors	1.181	0.684	2.037	0.597	0.551
Boyarsky, (C), 2021	Calcineurin inhibitors	1.011	0.614	1.663	0.043	0.966
Kamar 2021	Calcineurin inhibitors	1.173	0.683	2.014	0.577	0.564
Holden 2021	Calcineurin inhibitors	1.087	0.635	1.862	0.304	0.761
Sadioglu 2021	Calcineurin inhibitors	1.136	0.661	1.955	0.462	0.644
Kumar 2022	Calcineurin inhibitors	1.191	0.700	2.028	0.645	0.519
Caillard 2022	Calcineurin inhibitors	1.153	0.669	1.986	0.512	0.608
Mazzola 2021	Calcineurin inhibitors	1.137	0.653	1.982	0.454	0.649
Russo, 2021	Calcineurin inhibitors	1.130	0.658	1.940	0.444	0.657
Vaiciuniene, 2021	Calcineurin inhibitors	1.187	0.689	2.043	0.618	0.537
Chukwu, 2022	Calcineurin inhibitors	1.206	0.704	2.064	0.682	0.495
D'Offizi, 2021	Calcineurin inhibitors	1.029	0.605	1.751	0.105	0.916
Hod, 2021	Calcineurin inhibitors	1.136	0.653	1.977	0.452	0.651
Kantauskaite, 2021	Calcineurin inhibitors	1.135	0.657	1.961	0.454	0.650
Seija, 2022	Calcineurin inhibitors	1.162	0.665	2.028	0.527	0.598
Timmermann, 2021	Calcineurin inhibitors	1.034	0.604	1.771	0.121	0.904
		1.130	0.665	1.922	0.452	0.651

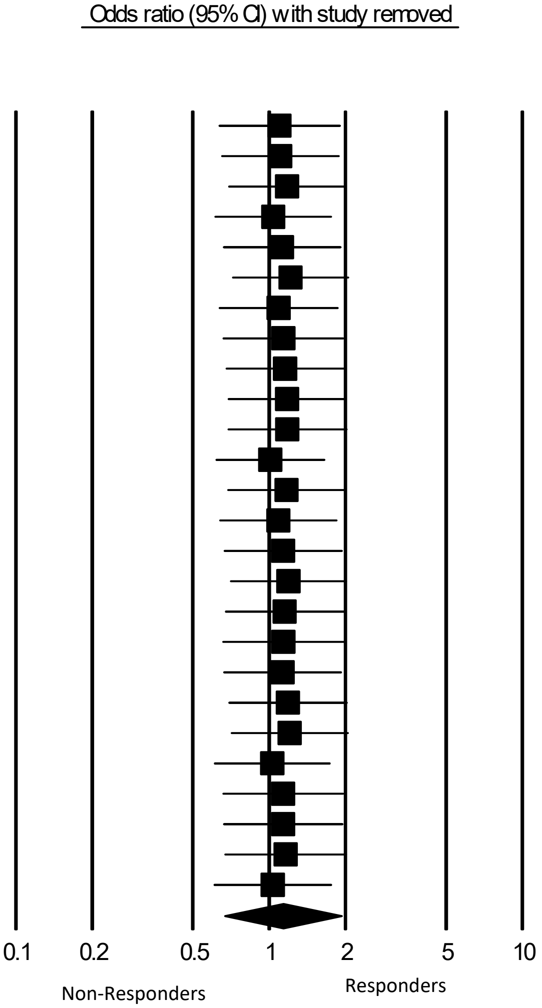

After the removal of one study, the significance of the point estimates is lost and this means that the heterogeneity is low and the removed study was causing the increased variability, the results are certain and robust.

Type of donor

Deceased donor status sensitivity

Study name	Subgroup within study	Statistics with study removed				
		Point	Lower limit	Upper limit	Z-Value	p-Value
Grupper, 2021	Deceased donor status	1.130	0.728	1.754	0.546	0.585
Benotmane, 2021	Deceased donor status	1.000	0.599	1.671	0.002	0.999
Reindl-Schwaighofer 2021	Deceased donor status	0.978	0.592	1.615	-0.088	0.930
Sadioglu 2021	Deceased donor status	0.875	0.641	1.195	-0.842	0.400
Caillard 2022	Deceased donor status	1.060	0.662	1.697	0.241	0.810
Erol 2021	Deceased donor status	0.972	0.616	1.533	-0.122	0.903
Debska-Slizien, 2021	Deceased donor status	0.986	0.617	1.575	-0.060	0.952
Chukwu, 2022	Deceased donor status	1.024	0.587	1.786	0.083	0.934
Hod, 2021	Deceased donor status	1.104	0.717	1.700	0.448	0.654
		1.005	0.651	1.552	0.025	0.980

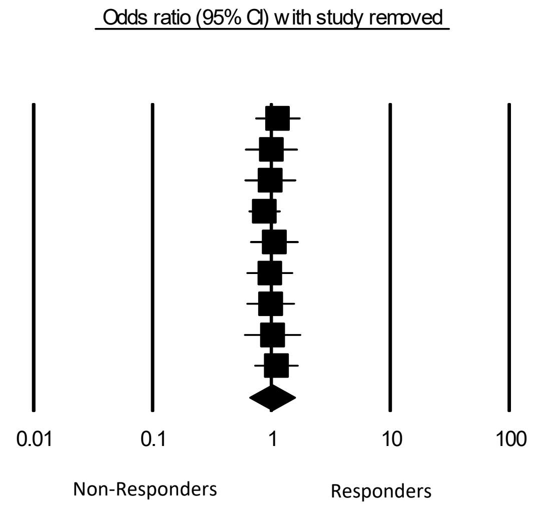

After the removal of one study, the significance of the point estimates is lost and this means that the heterogeneity is low and the removed study was causing the increased variability, the results are certain and robust.

mTor

mTOR inhibitors sensitivity

Study name	Subgroup within study	Statistics with study removed				
		Point	Lower limit	Upper limit	Z-Value	p-Value
Kamar, 2021	mTOR inhibitors	1.865	1.308	2.661	3.440	0.001
Peled, 2021	mTOR inhibitors	1.766	1.266	2.464	3.350	0.001
Hall, 2021	mTOR inhibitors	1.893	1.337	2.682	3.594	0.000
Boyarsky, 2021	mTOR inhibitors	1.743	1.270	2.391	3.441	0.001
Boyarsky (B), 2021	mTOR inhibitors	1.884	1.340	2.650	3.639	0.000
Shostak, 2021	mTOR inhibitors	2.027	1.485	2.766	4.455	0.000
Rozen-Zvi , 2021	mTOR inhibitors	1.946	1.377	2.750	3.773	0.000
Ben Zadok, 2021	mTOR inhibitors	1.802	1.274	2.549	3.330	0.001
Grupper, 2021	mTOR inhibitors	1.866	1.314	2.651	3.485	0.000
Rabinowich, 2021	mTOR inhibitors	1.885	1.325	2.683	3.522	0.000
Korth, 2021	mTOR inhibitors	1.890	1.342	2.661	3.643	0.000
Benotmane, 2021	mTOR inhibitors	1.821	1.271	2.609	3.269	0.001
Peled 2021	mTOR inhibitors	1.807	1.275	2.562	3.324	0.001
Kamar 2021	mTOR inhibitors	1.869	1.320	2.647	3.522	0.000
Holden 2021	mTOR inhibitors	1.863	1.321	2.628	3.544	0.000
Sadioglu 2021	mTOR inhibitors	1.869	1.327	2.634	3.575	0.000
Kumar 2022	mTOR inhibitors	1.894	1.344	2.667	3.653	0.000
Caillard 2022	mTOR inhibitors	1.896	1.335	2.692	3.576	0.000
Mazzola 2021	mTOR inhibitors	1.929	1.363	2.731	3.705	0.000
Russo, 2021	mTOR inhibitors	1.884	1.329	2.671	3.558	0.000
Davidov, 2021	mTOR inhibitors	2.041	1.485	2.807	4.392	0.000
Hod, 2021	mTOR inhibitors	1.881	1.328	2.662	3.561	0.000
Kantauskaite, 2021	mTOR inhibitors	1.812	1.281	2.562	3.361	0.001
Seija, 2022	mTOR inhibitors	1.758	1.235	2.502	3.132	0.002
Timmermann, 2021	mTOR inhibitors	1.880	1.335	2.646	3.617	0.000
		1.870	1.335	2.619	3.639	0.000

Odds ratio (95% CI) with study removed

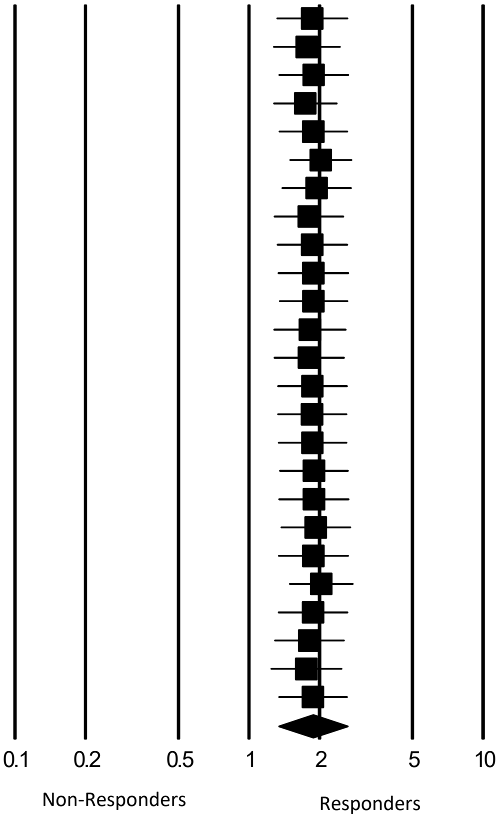

After removing one study, the p- value did not lose the significance of the point estimates and this means that there is a high heterogeneity among the studies, and this may lead to a decreased certainty of the results due to wide variability between studies.

Figure S3: Funnel plots

Age

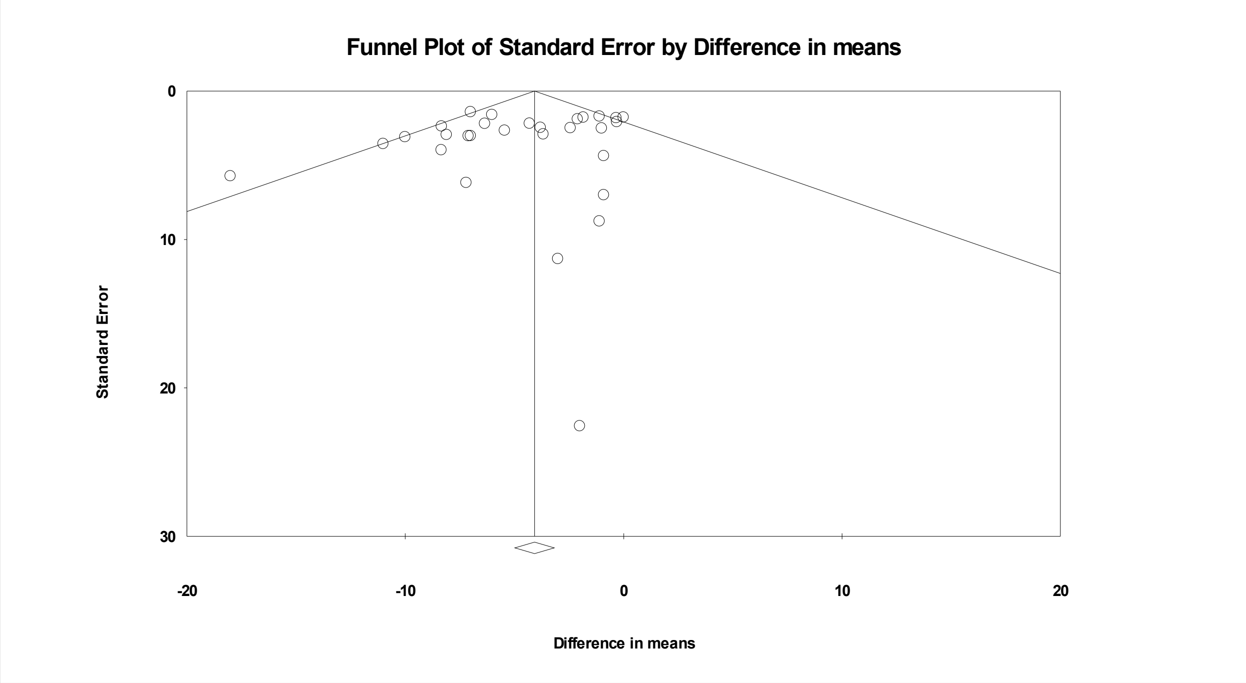

The funnel plot is approximately similar (no publication bias exists), only one study have high standard error.

Antimetabolites

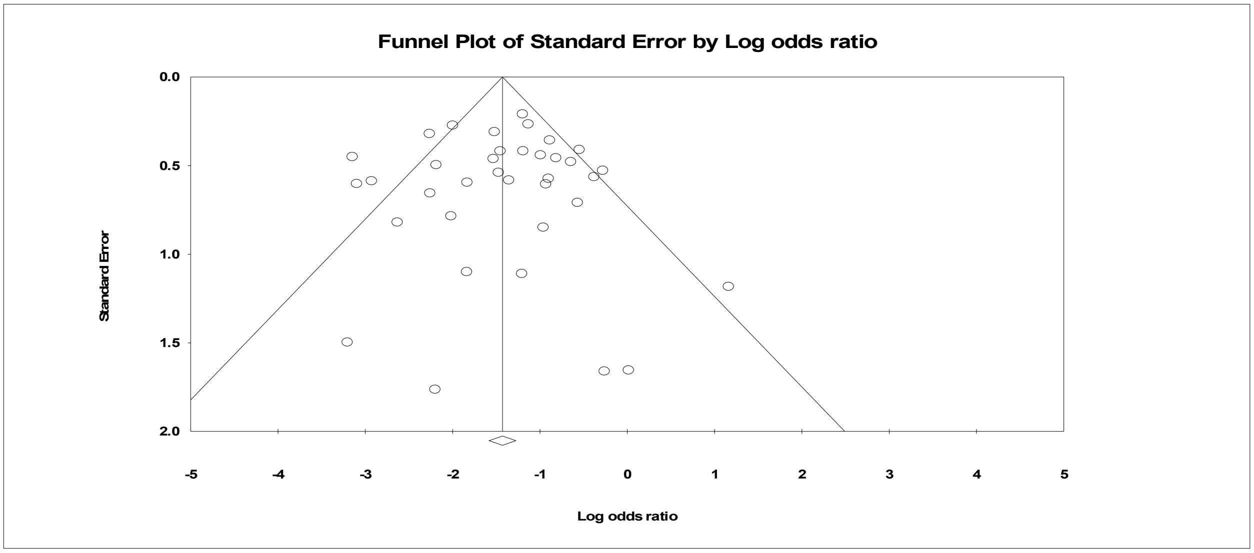

The graph is approximately similar (no publication bias exists)

BMI

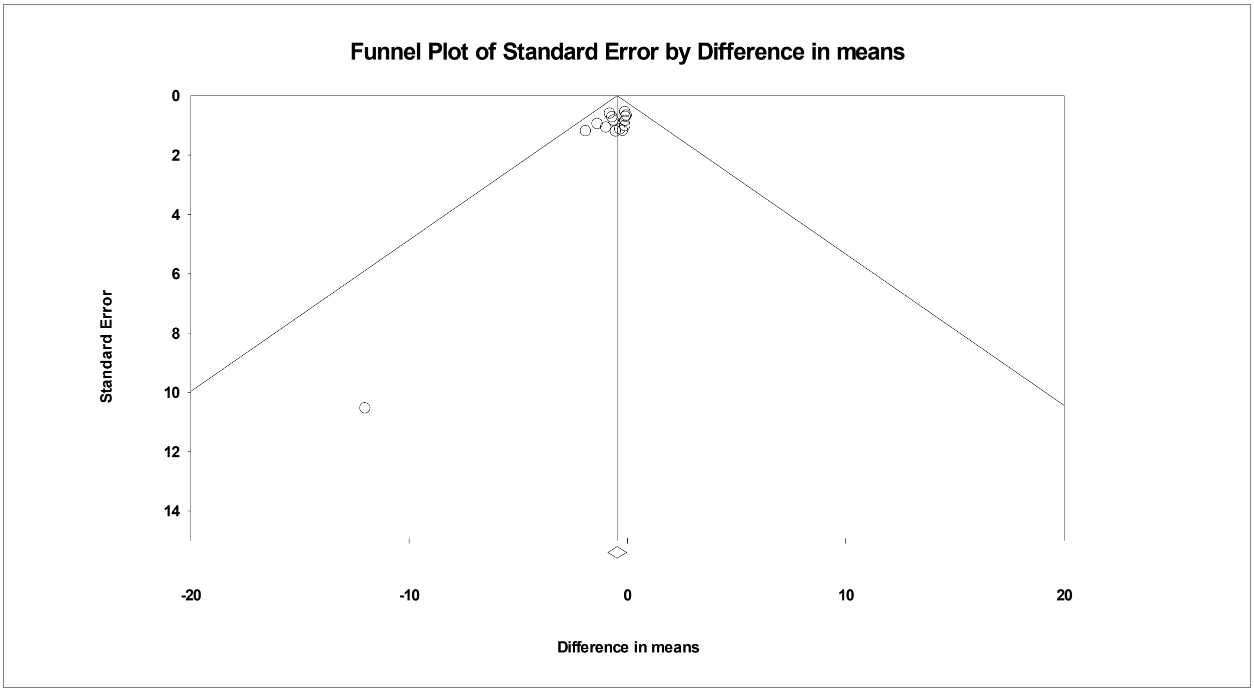

All the studies taking BMI into consideration as an important risk factor for vaccine response are precise and have low standard error, all the studies are situated on the upper part of the funnel plot except one study that has a high standard error.

Time from transplant

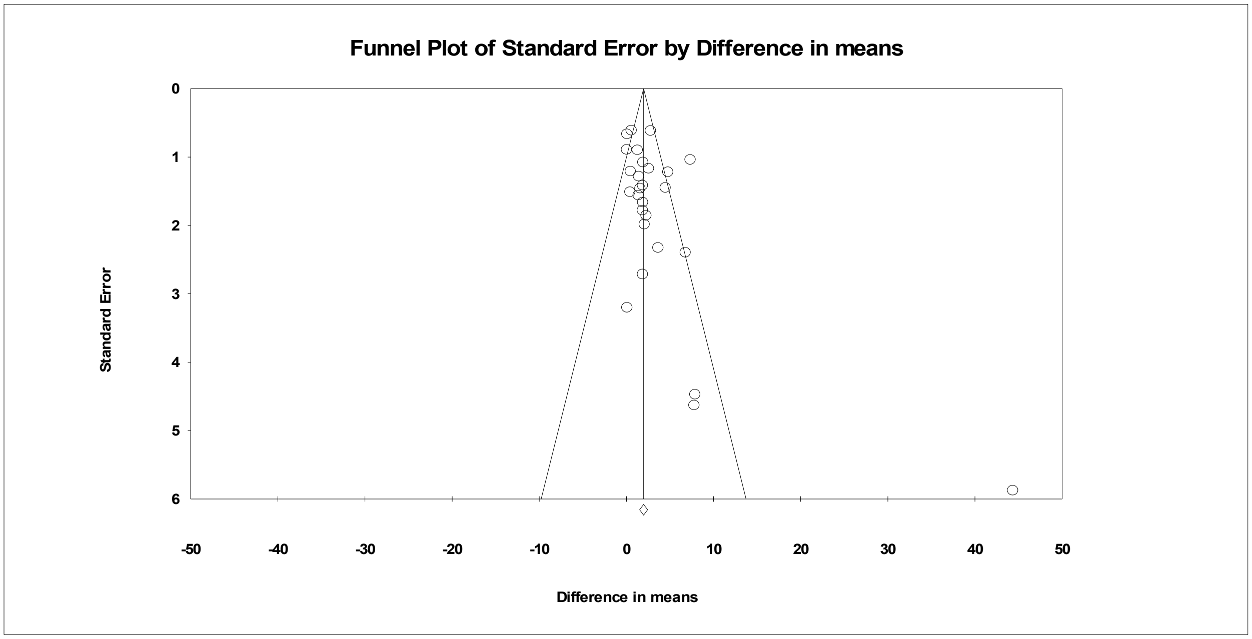

The funnel plot is approximately similar (no publication bias exists)

Calcineurin inhibitors

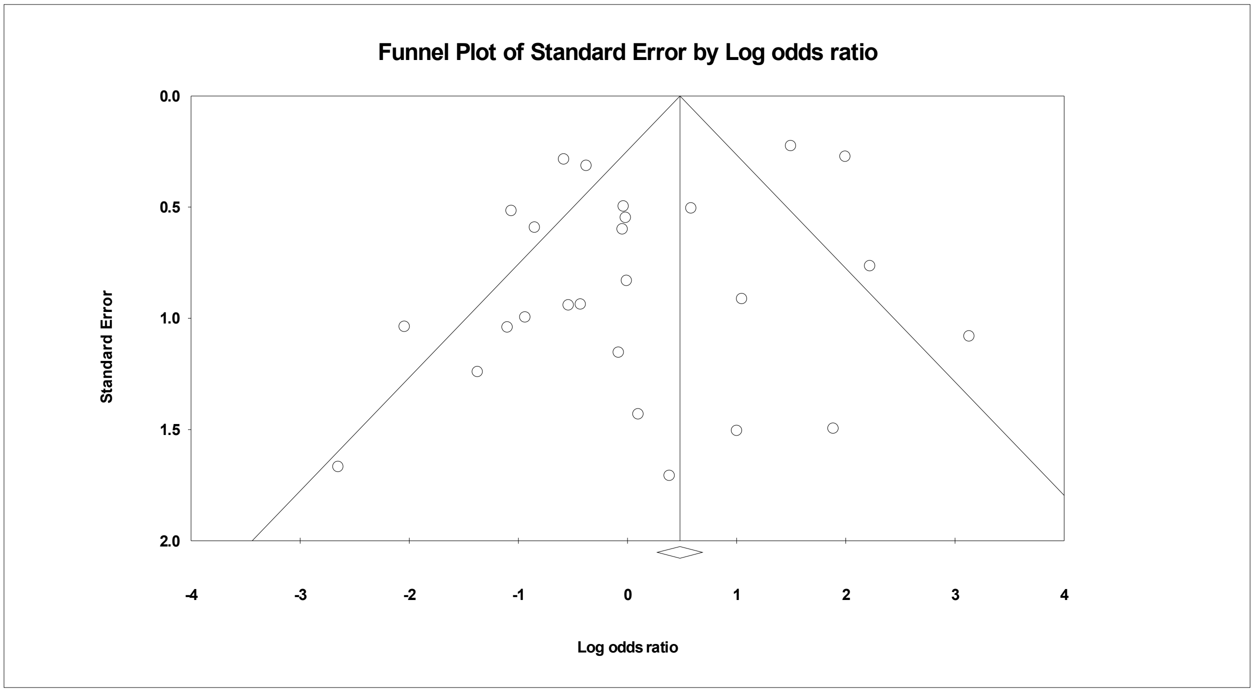

The funnel plot is approximately similar (no publication bias exists)

Gender (males)

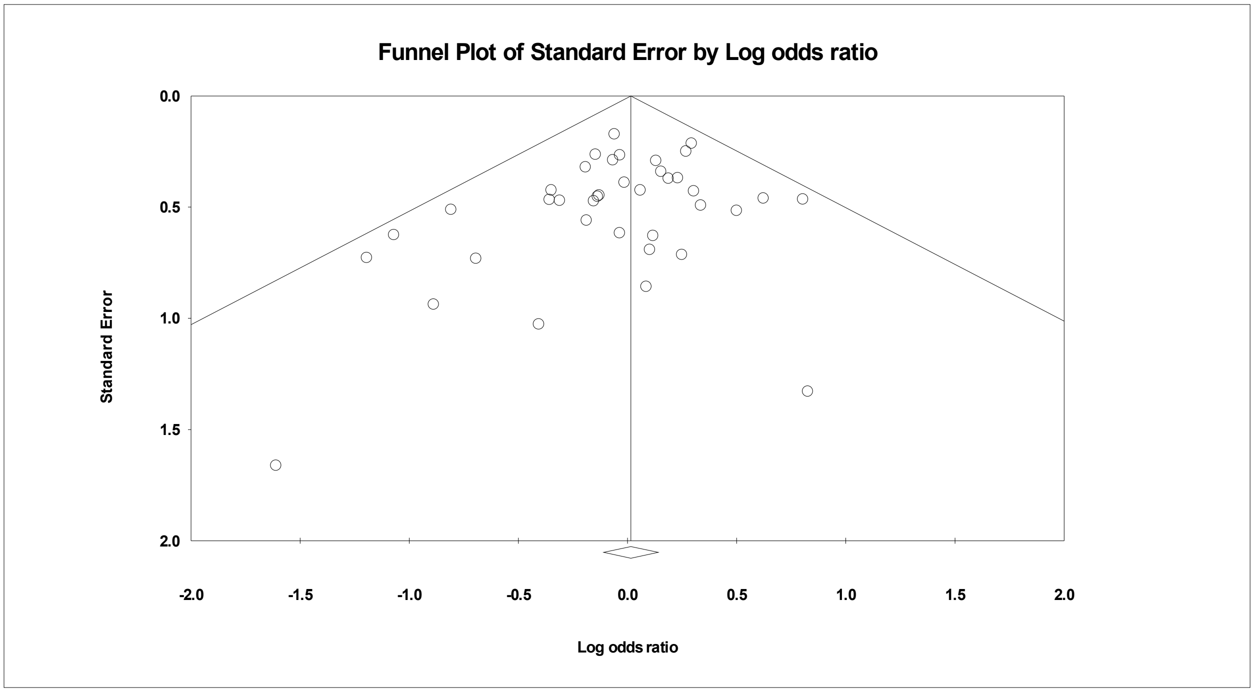

The funnel plot is approximately similar (no publication bias exists)

mTOR inhibitors

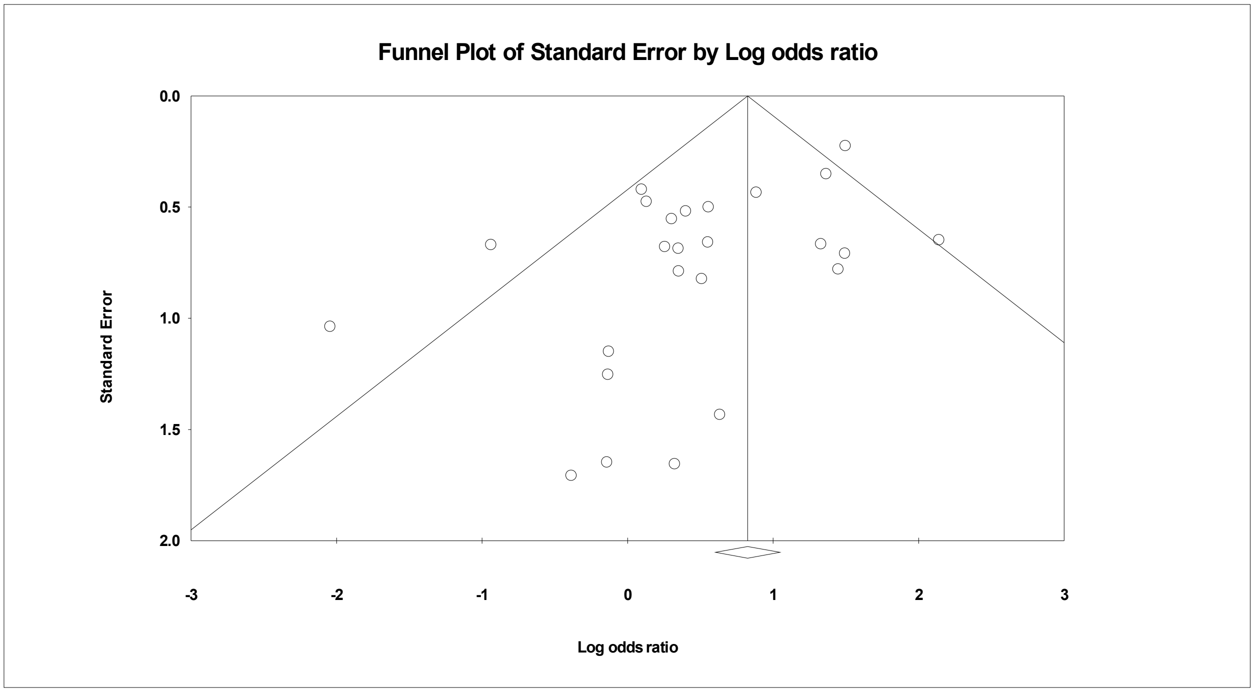

The funnel plot is not quite similar and this may be due to the presence of a publication bias or a difference in methodological quality between studies used in this plot, these studies may differ in the sample size and this leads to higher heterogeneity between the studies.
