# Supplementary material for: Humoral and Cellular Immunity following Five Doses of COVID-19 Vaccines in Solid Organ Transplant Recipients: A Systematic Review and Meta-Analysis
Source: Vaccines (Basel). 2023 Jun 27;11(7):1166. doi: 10.3390/vaccines11071166 (PMC10384009; doi:10.3390/vaccines11071166)
Supplement: Supplementary file 1 [file vaccines-11-01166-s001.zip › File S1 Research Strategy.pdf]

Key Words:

- Organ Transplant Recipients , Solid Organ transplant patients , humoral and cellular Immunity , Antibodies titers , Antibody Response , Impaired immunity , organ transplantation , Lung, Heart ,Kidney ,spleen transplantation , BNT162b2 (BioNTech/Pfizer), Ad26.COV2.S (Janssen COVID-19 vaccine), mRNA-1273 (moderna), ChAdOx1 nCoV-19(Oxford/AstraZeneca), **inactivated whole-virus SARS-CoV-2 vaccine, CoronaVac® (Sinovac Biotech Ltd.),**

Search strategy :

A systematic literature search was performed using PubMed, EMBASE, Clinicaltrials.gov and web of science to identify the ongoing and completed clinical Trials of humoral and cellular immunity following covid-19 vaccines in organ transplant recipients and retrieved all studies identified from 1/9/2021 to 1/1/2023

We Included any Solid Organ Transplant recipient over 18 years of age who receive one or two or three or four doses of any type of Covid-19 vaccines & SOT who on immunosuppressant medication . Randomized Clinical Trials , Cohorts , cases series and case reports are included as well.

We Excluded immunocompromised, dialysis & Patients who had recovered from SARS-CoV- 2 or had active infection at the time of the vaccination or up to 7 days after the second vaccine dose ,
and Patients with a positive SARS-CoV-2 polymerase chain reaction test before or after the first vaccination and during the first week after the second vaccination .

(((((((((((((((((((((((((((((Humoral and cellular Immunity) OR (Antibodies titers)) OR (Antibody Response))) OR (impaired immunity)) AND (Coronavirus)) OR (covid19)) OR (covid2019)) OR (SARS2)) OR (COV-19)) OR (Novel covid))) AND (Organ Transplant Recipients)) OR (Solid Organ transplant patients)) OR (organ transplantation)) OR (Liver Transplant Recipients)) OR (Kidney Transplant Recipients)) OR (Heart Transplant Recipients)) AND (covid-19 vaccine)) OR (BNT162b2)) OR (BioNTech/Pfizer)) OR (Ad26.COV2.S)) OR (Janssen COVID-19 vaccine)) OR (mRNA-1273)) OR (moderna)) OR (ChAdOx1 nCoV-19)) OR (Oxford/AstraZeneca)) OR (inactivated whole-virus SARS-CoV-2 vaccine)) OR (CoronaVac)) OR (Sinovac Biotech) from 2021/8/1 – 1/1/2023

((((((((("humoral"[All Fields] OR "humorally"[All Fields] OR "humoural"[All Fields]) AND ("immunity, cellular"[MeSH Terms] OR ("immunity"[All Fields] AND "cellular"[All Fields]) OR "cellular immunity"[All Fields] OR ("cellular"[All Fields] AND "immunity"[All Fields]))) OR (("antibodie"[All Fields] OR "antibodies"[MeSH Terms] OR "antibodies"[All Fields] OR "antibody s"[All Fields] OR "antibodys"[All Fields] OR "immunoglobulins"[MeSH Terms] OR "immunoglobulins"[All Fields] OR "antibody"[All Fields]) AND ("titer"[All Fields] OR "titered"[All Fields] OR "titering"[All Fields] OR "titers"[All Fields] OR "titre"[All Fields] OR "titred"[All Fields] OR "titres"[All Fields])) OR ("antibody formation"[MeSH Terms] OR ("antibody"[All Fields] AND "formation"[All Fields]) OR "antibody formation"[All Fields] OR ("antibody"[All Fields] AND "response"[All Fields]) OR "antibody

response"[All Fields]) OR (("impair"[All Fields] OR "impaired"[All Fields] OR "impairement"[All Fields] OR "impairements"[All Fields] OR "impairing"[All Fields] OR "impairment"[All Fields] OR "impairments"[All Fields] OR "impairs"[All Fields]) AND ("immune"[All Fields] OR "immuned"[All Fields] OR "immunes"[All Fields] OR "immunisation"[All Fields] OR "vaccination"[MeSH Terms] OR "vaccination"[All Fields] OR "immunization"[All Fields] OR "immunization"[MeSH Terms] OR "immunisations"[All Fields] OR "immunizations"[All Fields] OR "immunise"[All Fields] OR "immunised"[All Fields] OR "immuniser"[All Fields] OR "immunisers"[All Fields] OR "immunising"[All Fields] OR "immunities"[All Fields] OR "immunity"[MeSH Terms] OR "immunity"[All Fields] OR "immunization s"[All Fields] OR "immunize"[All Fields] OR "immunized"[All Fields] OR "immunizer"[All Fields] OR "immunizers"[All Fields] OR "immunizes"[All Fields] OR "immunizing"[All Fields]))) AND ("coronavirus"[MeSH Terms] OR "coronavirus"[All Fields] OR "coronaviruses"[All Fields])) OR ("covid 19"[MeSH Terms] OR "covid 19"[All Fields] OR "covid19"[All Fields]) OR "covid2019"[All Fields] OR ("sars cov 2"[MeSH Terms] OR "sars cov 2"[All Fields] OR "sars2"[All Fields]) OR "COV-19"[All Fields] OR (("novel"[All Fields] OR "novel s"[All Fields] OR "novels"[All Fields]) AND ("sars cov 2"[MeSH Terms] OR "sars cov 2"[All Fields] OR "covid"[All Fields] OR "covid 19"[MeSH Terms] OR "covid 19"[All Fields]))) AND (("transplants"[MeSH Terms] OR "transplants"[All Fields] OR ("organ"[All Fields] AND "transplant"[All Fields]) OR "organ transplant"[All Fields] OR "organ transplantation"[MeSH Terms] OR ("organ"[All Fields] AND "transplantation"[All Fields]) OR "organ transplantation"[All Fields] OR ("organ"[All Fields] AND "transplant"[All Fields])) AND ("recipient"[All Fields] OR "recipient s"[All Fields] OR "recipients"[All Fields]))) OR (("solid"[All Fields] OR "solid s"[All Fields] OR "solids"[All Fields]) AND ("transplants"[MeSH Terms] OR "transplants"[All Fields] OR ("organ"[All Fields] AND "transplant"[All Fields]) OR "organ transplant"[All Fields] OR "organ transplantation"[MeSH Terms] OR ("organ"[All Fields] AND "transplantation"[All Fields]) OR "organ transplantation"[All Fields] OR ("organ"[All Fields] AND "transplant"[All Fields])) AND ("patient s"[All Fields] OR "patients"[MeSH Terms] OR "patients"[All Fields] OR "patient"[All Fields] OR "patients s"[All Fields])) OR ("organ transplantation"[MeSH Terms] OR ("organ"[All Fields] AND "transplantation"[All Fields]) OR "organ transplantation"[All Fields]) OR (("liver transplantation"[MeSH Terms] OR ("liver"[All Fields] AND "transplantation"[All Fields]) OR "liver transplantation"[All Fields] OR ("liver"[All Fields] AND "transplant"[All Fields]) OR "liver transplant"[All Fields]) AND ("recipient"[All Fields] OR "recipient s"[All Fields] OR "recipients"[All Fields])) OR (("kidney transplantation"[MeSH Terms] OR ("kidney"[All Fields] AND "transplantation"[All Fields]) OR "kidney transplantation"[All Fields] OR ("kidney"[All Fields] AND "transplant"[All Fields]) OR "kidney transplant"[All Fields]) AND ("recipient"[All Fields] OR "recipient s"[All Fields] OR "recipients"[All Fields])) OR (("heart transplantation"[MeSH Terms] OR ("heart"[All Fields] AND "transplantation"[All Fields]) OR "heart transplantation"[All Fields] OR ("heart"[All Fields] AND "transplant"[All Fields]) OR "heart transplant"[All Fields]) AND ("recipient"[All Fields] OR "recipient s"[All Fields] OR "recipients"[All Fields]))) AND ("covid 19 vaccines"[MeSH Terms] OR ("covid 19"[All Fields] AND "vaccines"[All Fields]) OR "covid 19 vaccines"[All Fields] OR "covid 19 vaccine"[All Fields])) OR ("bnt162 vaccine"[MeSH Terms] OR ("bnt162"[All Fields] AND "vaccine"[All Fields]) OR "bnt162 vaccine"[All Fields] OR "bnt162b2"[All Fields]) OR "biontech pfizer"[All Fields] OR ("ad26covs1"[MeSH Terms] OR "ad26covs1"[All Fields] OR "ad26 cov2 s"[All Fields]) OR ("Janssen"[All Fields] AND ("covid 19 vaccines"[MeSH Terms] OR ("covid 19"[All Fields] AND "vaccines"[All Fields]) OR "covid 19 vaccines"[All Fields] OR "covid 19 vaccine"[All Fields])) OR ("2019 ncov vaccine mrna 1273"[MeSH Terms] OR ("2019 ncov"[All Fields] AND "vaccine"[All Fields] AND "mrna 1273"[All Fields]) OR "2019 ncov vaccine mrna 1273"[All Fields] OR "mrna 1273"[All Fields]) OR "moderna"[All Fields] OR ("chadox1 ncov 19"[MeSH Terms] OR ("chadox1"[All Fields] AND "ncov 19"[All Fields]) OR "chadox1

ncov 19"[All Fields] OR "chadox1 ncov 19"[All Fields]) OR "oxford astrazeneca"[All Fields] OR (("inactivate"[All Fields] OR "inactivated"[All Fields] OR "inactivates"[All Fields] OR "inactivating"[All Fields] OR "inactivation"[All Fields] OR "inactivations"[All Fields] OR "inactivator"[All Fields] OR "inactivators"[All Fields] OR "inactived"[All Fields]) AND "whole-virus"[All Fields] AND ("covid 19 vaccines"[MeSH Terms] OR ("covid 19"[All Fields] AND "vaccines"[All Fields]) OR "covid 19 vaccines"[All Fields] OR "sars cov 2 vaccine"[All Fields])) OR "CoronaVac"[All Fields] OR ("Sinovac"[All Fields] AND ("biotech"[All Fields] OR "biotech s"[All Fields] OR "biotechs"[All Fields]))) AND ((ffrft[Filter]) AND (randomizedcontrolledtrial[Filter]) AND (2021/8/1:2022/1/1[pdat]))

Translations

Humoral: "humoral"[All Fields] OR "humorally"[All Fields] OR "humoural"[All Fields]

cellular Immunity: "immunity, cellular"[MeSH Terms] OR ("immunity"[All Fields] AND "cellular"[All Fields]) OR "cellular immunity"[All Fields] OR ("cellular"[All Fields] AND "immunity"[All Fields])

Antibodies: "antibodie"[All Fields] OR "antibodies"[MeSH Terms] OR "antibodies"[All Fields] OR "antibody's"[All Fields] OR "antibodys"[All Fields] OR "immunoglobulins"[MeSH Terms] OR "immunoglobulins"[All Fields] OR "antibody"[All Fields]

titers: "titer"[All Fields] OR "titered"[All Fields] OR "titering"[All Fields] OR "titers"[All Fields] OR "titre"[All Fields] OR "titred"[All Fields] OR "titres"[All Fields]

Antibody Response: "antibody formation"[MeSH Terms] OR ("antibody"[All Fields] AND "formation"[All Fields]) OR "antibody formation"[All Fields] OR ("antibody"[All Fields] AND "response"[All Fields]) OR "antibody response"[All Fields]

impaired: "impair"[All Fields] OR "impaired"[All Fields] OR "impairement"[All Fields] OR "impairements"[All Fields] OR "impairing"[All Fields] OR "impairment"[All Fields] OR "impairments"[All Fields] OR "impairs"[All Fields]

immunity: "immune"[All Fields] OR "immuned"[All Fields] OR "immunes"[All Fields] OR "immunisation"[All Fields] OR "vaccination"[MeSH Terms] OR "vaccination"[All Fields] OR "immunization"[All Fields] OR "immunization"[MeSH Terms] OR "immunisations"[All Fields] OR "immunizations"[All Fields] OR "immunise"[All Fields] OR "immunised"[All Fields] OR "immuniser"[All Fields] OR "immunisers"[All Fields] OR "immunising"[All Fields] OR "immunities"[All Fields] OR "immunity"[MeSH Terms] OR "immunity"[All Fields] OR "immunization's"[All Fields] OR "immunize"[All Fields] OR "immunized"[All Fields] OR "immunizer"[All Fields] OR "immunizers"[All Fields] OR "immunizes"[All Fields] OR "immunizing"[All Fields]

Coronavirus: "coronavirus"[MeSH Terms] OR "coronavirus"[All Fields] OR "coronaviruses"[All Fields]

covid19: "covid-19"[MeSH Terms] OR "covid-19"[All Fields] OR "covid19"[All Fields]

SARS2: "sars-cov-2"[MeSH Terms] OR "sars-cov-2"[All Fields] OR "sars2"[All Fields]

Novel: "novel"[All Fields] OR "novel's"[All Fields] OR "novels"[All Fields]

covid: "sars-cov-2"[MeSH Terms] OR "sars-cov-2"[All Fields] OR "covid"[All Fields] OR "covid-19"[MeSH Terms] OR "covid-19"[All Fields]

Organ Transplant: "transplants"[MeSH Terms] OR "transplants"[All Fields] OR ("organ"[All Fields] AND "transplant"[All Fields]) OR "organ transplant"[All Fields] OR "organ transplantation"[MeSH Terms] OR ("organ"[All Fields] AND "transplantation"[All Fields]) OR "organ transplantation"[All Fields] OR ("organ"[All Fields] AND "transplant"[All Fields])

Recipients: "recipient"[All Fields] OR "recipient's"[All Fields] OR "recipients"[All Fields]

Solid: "solid"[All Fields] OR "solid's"[All Fields] OR "solids"[All Fields]

Organ transplant: "transplants"[MeSH Terms] OR "transplants"[All Fields] OR ("organ"[All Fields] AND "transplant"[All Fields]) OR "organ transplant"[All Fields] OR "organ transplantation"[MeSH Terms] OR ("organ"[All Fields] AND "transplantation"[All Fields]) OR "organ transplantation"[All Fields] OR ("organ"[All Fields] AND "transplant"[All Fields])

patients: "patient's"[All Fields] OR "patients"[MeSH Terms] OR "patients"[All Fields] OR "patient"[All Fields] OR "patients's"[All Fields]

organ transplantation: "organ transplantation"[MeSH Terms] OR ("organ"[All Fields] AND "transplantation"[All Fields]) OR "organ transplantation"[All Fields]

Liver Transplant: "liver transplantation"[MeSH Terms] OR ("liver"[All Fields] AND "transplantation"[All Fields]) OR "liver transplantation"[All Fields] OR ("liver"[All Fields] AND "transplant"[All Fields]) OR "liver transplant"[All Fields]

Recipients: "recipient"[All Fields] OR "recipient's"[All Fields] OR "recipients"[All Fields]

Kidney Transplant: "kidney transplantation"[MeSH Terms] OR ("kidney"[All Fields] AND "transplantation"[All Fields]) OR "kidney transplantation"[All Fields] OR ("kidney"[All Fields] AND "transplant"[All Fields]) OR "kidney transplant"[All Fields]

Recipients: "recipient"[All Fields] OR "recipient's"[All Fields] OR "recipients"[All Fields]

Heart Transplant: "heart transplantation"[MeSH Terms] OR ("heart"[All Fields] AND "transplantation"[All Fields]) OR "heart transplantation"[All Fields] OR ("heart"[All Fields] AND "transplant"[All Fields]) OR "heart transplant"[All Fields]

Recipients: "recipient"[All Fields] OR "recipient's"[All Fields] OR "recipients"[All Fields]

covid-19 vaccine: "covid-19 vaccines"[MeSH Terms] OR ("covid-19"[All Fields] AND "vaccines"[All Fields]) OR "covid-19 vaccines"[All Fields] OR "covid 19 vaccine"[All Fields]

BNT162b2: "bnt162 vaccine"[MeSH Terms] OR ("bnt162"[All Fields] AND "vaccine"[All Fields]) OR "bnt162 vaccine"[All Fields] OR "bnt162b2"[All Fields]

Ad26.COV2.S: "ad26covs1"[MeSH Terms] OR "ad26covs1"[All Fields] OR "ad26 cov2 s"[All Fields]

COVID-19 vaccine: "covid-19 vaccines"[MeSH Terms] OR ("covid-19"[All Fields] AND "vaccines"[All Fields]) OR "covid-19 vaccines"[All Fields] OR "covid 19 vaccine"[All Fields]

mRNA-1273: "2019-ncov vaccine mrna-1273"[MeSH Terms] OR ("2019-ncov"[All Fields] AND "vaccine"[All Fields] AND "mrna-1273"[All Fields]) OR "2019-ncov vaccine mrna-1273"[All Fields] OR "mrna 1273"[All Fields]

ChAdOx1 nCoV-19: "chadox1 ncov-19"[MeSH Terms] OR ("chadox1"[All Fields] AND "ncov-19"[All Fields]) OR "chadox1 ncov-19"[All Fields] OR "chadox1 ncov 19"[All Fields]

inactivated: "inactivate"[All Fields] OR "inactivated"[All Fields] OR "inactivates"[All Fields] OR "inactivating"[All Fields] OR "inactivation"[All Fields] OR "inactivations"[All Fields] OR "inactivator"[All Fields] OR "inactivators"[All Fields] OR "inactived"[All Fields]

SARS-CoV-2 vaccine: "covid-19 vaccines"[MeSH Terms] OR ("covid-19"[All Fields] AND "vaccines"[All Fields]) OR "covid-19 vaccines"[All Fields] OR "sars cov 2 vaccine"[All Fields]

Biotech: "biotech"[All Fields] OR "biotech's"[All Fields] OR "biotechs"[All Fields]
